# The Par-PrkC Polarity Complex Is Required for Cilia Growth in Zebrafish Photoreceptors

**DOI:** 10.1371/journal.pone.0104661

**Published:** 2014-08-21

**Authors:** Bryan L. Krock, Brian D. Perkins

**Affiliations:** 1 Department of Biology, Texas A&M University, College Station, Texas, United States of America; 2 Department of Ophthalmic Research, Cole Eye Institute, Cleveland Clinic Foundation, Cleveland, Ohio, United States of America; Texas A&M International University, United States of America

## Abstract

Specification and development of the apical membrane in epithelial cells requires the function of polarity proteins, including Pard3 and an atypical protein kinase C (PrkC). Many epithelial cells possess microtubule-based organelles, known as cilia, that project from their apical surface and the membrane surrounding the cilium is contiguous with the apical cell membrane. Although cilia formation in cultured cells required Pard3, the *in* *vivo* requirement for Pard3 in cilia development remains unknown. The vertebrate photoreceptor outer segment represents a highly specialized cilia structure in which to identify factors necessary for apical and ciliary membrane formation. Pard3 and PrkC localized to distinct domains within vertebrate photoreceptors. Using partial morpholino knockdown, photo-morpholinos, and pharmacological approaches, the function of Pard3 and PrkC were found to be required for the formation of both the apical and ciliary membrane of vertebrate photoreceptors. Inhibition of Pard3 or PrkC activity significantly reduced the size of photoreceptor outer segments and resulted in mislocalization of rhodopsin. Suppression of Pard3 or PrkC also led to a reduction in cilia size and cilia number in Kupffer’s Vesicle, which resulted in left-right asymmetry defects. Thus, the Par-PrkC complex functions in cilia formation *in* *vivo* and this likely reflects a general role in specifying non-ciliary and ciliary compartments of the apical domain.

## Introduction

Cilia are hair-like structures that protrude from the apical surface of almost all vertebrate cells, including polarized epithelial cells. These cilia serve diverse functions, including sensory reception, motility-driven fluid flow, and signaling [1]–[3]. Within the cilium, the microtubule-based axoneme projects from a basal body anchored at the apical surface. This structure is surrounded by a ciliary membrane that is contiguous with the plasma membrane. The formation and continued maintenance of cilia requires intraflagellar transport (IFT), which refers to the bidirectional movement of IFT particles along the axoneme. Anterograde movement up the cilium is controlled by two kinesin motors, the heterotrimeric kinesin-II and the homodimeric Kif17 [4]–[7], while retrograde movement is controlled by the cytoplasmic dynein-2 complex [8]–[11].

Ciliogenesis initiates with the positioning of the centrosome/basal body at the apical surface of epithelial cells. While the precise mechanisms controlling this action have not been fully elucidated, components of the cytoskeleton as well as determinants of cell polarity have been implicated. In primary cultures of multiciliated epithelial cells, basal body docking coincided with the assembly of an actin web at the apical surface [12], [13]. Pharmacological inhibition of actin assembly prevented the apical accumulation of basal bodies and blocked ciliogenesis [12]. Although disruption of microtubules did not affect basal body migration in cultured quail oviducts [14], a dynamic microtubule reorganization was required for apical positioning of centrosomes and ciliogenesis within the zebrafish neural tube [15]. This microtubule reorganization and centrosome migration was also dependent upon the polarity protein Pard3 [15].

Pard3 is one of three key polarity determinants, along with Par6, and protein kinase C iota (PrkCi; formerly known as aPKCλ), that form a heterotrimeric complex responsible for regulating apico-basal polarity in animal cells [16]. In zebrafish, three *pard3* paralogs exist: *pard3a*, *pard3ba*, and *pard3bb*. We have focused on *pard3a*, which has been the subject of previous studies [15], [17], and will be referred to herein as *pard3*.

Pard3, Par6, and PrkCi function in the maturation of tight junctions and adherens junctions but also determine the apical membrane domain (for reviews see [18], [19]). Par6 and PrkCi physically interact through their N-terminal domains, while PrkCi physically interacts with Pard3 in a dynamic fashion. Following cell-cell contact, Pard3 recruits the Par6-PrkCi complex to contact sites, upon which tight junctions eventually form [20]–[22]. Following tight junction formation, growing evidence suggests that the PrkCi-Par6 complex dissociates from Pard3 and moves to the apical surface, where it localizes with other apical factors like Cdc42 and the Crumbs (Crb) complex [21], [23]–[26]. Elimination of any of these proteins or mutations that disrupt their interactions reduces the size of the apical membrane. Thus, while Pard3, Par6, and PrkCi all regulate apical domain size, only the Par6-PrkCi complex migrates to the apical surface to interact with the Crb complex [18].

The relationship between apical membrane size and ciliogenesis has been extensively studied in vertebrate photoreceptors. Vertebrate photoreceptors exhibit strong differences, both molecularly and morphologically, along the apical-basal axis. The presynaptic machinery localizes at the basal end of the cell while the apical domain consists of the distal part of the inner segment and the large outer segment. The photoreceptor outer segment is considered a modified “sensory cilium” [27] and extends from the apical surface of the inner segment. Numerous studies have implicated the Crb complex as a regulator of apical identity and outer segment size in both *Drosophila* and vertebrates [26], [28]–[31]. Loss of the zebrafish ortholog *crb2b* results in a reduction of apical domain size and loss of outer segments [28], while mutation of Crumbs homolog 1 (CRB1) causes early vision loss and photoreceptor degeneration [32], [33]. Overexpression of the intracellular domain of Crb2a in zebrafish photoreceptors leads to an expansion of the outer segments, supporting a role for the Crb complex in positively regulating the size of the apical domain, including cilia [29]. The role other apical determinants play in ciliogenesis remains less clear. Zebrafish lacking either PrkCi or the MAGUK protein Nagie Oko do not exhibit obvious cilia defects in the retina, pronephros, or otic vesicle [28]. In contrast, Pard3, Par6 and PrkCi localize to cilia in cultured Madin Darby canine kidney (MDCK) cells and suppression of Pard3 or PrkCi by siRNA blocked ciliogenesis in cell culture [34], [35]. Cilia growth in the neural tube was reported to be delayed, but not blocked, following suppression of zebrafish *pard3* by morpholinos [15] but a role for *pard3* in photoreceptor ciliogenesis was not investigated in a previous report [17]. Thus, it remains poorly understood whether the role of Crb in ciliogenesis is independent of other factors *in* *vivo*, or if other apical proteins, such as Pard3 and PrkCi, also play a role.

To better understand the role of apical identity proteins in ciliogenesis, we examined zebrafish photoreceptors following suppression of Pard3 or PrkCi. Partial suppression of these proteins by multiple experimental approaches dramatically reduced the size of the apical domain in photoreceptors and reduced cilia growth, resulting in smaller outer segments and mislocalization of rhodopsin. Our analysis suggests a general role for apical identity proteins in cilia formation *in* *vivo* and that cilia growth depends, in part, on the size of the apical membrane.

## Materials and Methods

### Nomenclature

In this paper, Pard3 (also known as Par3, Baz, Pard3a, or ASIP) will be referred to as Pard3. PrkCi (also known as aPKC lambda/iota or aPKCλ) will be referred to as PrkCi. PrkCz (also known as aPKC zeta or aPKCζ) will be referred to as PrkCz. The term PrkC will refer to the combined activity of PrkCi and PrkCz in photoreceptors.

### Ethics Statement

Wild-type zebrafish of a mixed AB-Ekkwill strain were housed, bred and staged according to standard procedures [36]. This study was carried out in strict accordance with the recommendations in the Guide for the Care and Use of Laboratory Animals of the National Institutes of Health. The protocol was approved by the Institutional Animal Care and Use Committees at Texas A&M University (Protocol Number: 2011-62) and the Cleveland Clinic (Protocol number: 2012-0853).

### Morpholino microinjection and design

One to two cell stage zebrafish embryos were injected as described [37]. All morpholinos (MOs) and photo-morpholinos (photo-MOs) were synthesized by Gene Tools, LLC. (Philomath, OR). The *pard3* MO1 (5′ TCAAAGGCTCCCGTGCTCTGGTGTC 3′) was injected at 1.5–2.0 ng per embryo and was and described and validated previously [15], [17]. The *pard3* mismatch MO (5′ TCAATTACTCCCGTGAACTGTTGTC 3′) contains six mismatches to the *pard3* MO1 sequence and was injected at 3.5 ng per embryo. Both morpholinos were designed as translation-blocking morpholinos and targeted the 5′ UTR of the *pard3* mRNA. The *prkci* morpholino sequence (5′ TGTCCCGCAGCGTGGGCATTATGGA 3′) and *prkcz* morpholino (5′ GATCCGTTACTGACAGGCATTATA 3′) were designed to inhibit translation and were used at 5 ng and 8 ng per embryo, respectively, as previously described [38], [39].

Sense photo-morpholinos (S-photo-MOs [40]) for *pard3* (5′ ACACCAGAGCApGGGAGCC), *prkci* (5′ ATAATGCCCApGCTGCGGGA 3′) and *prkcz* (5′ TATAATGCCTGpCAGTAACGGAT 3′) incorporate a photo-sensitive subunit (p) near the middle of the oligonucleotide, which can be cleaved by UV-light. S-photo-MOs can hybridize in a complementary fashion to the standard unmodified MOs, thereby permitting normal gene expression. Upon illumination by UV light, the photo-MO is cleaved and the standard MO is then free to bind the target RNA and block translation. Photo-MOs were mixed with their complementary MOs in 1∶1 or 0.9∶1.0 molar ratios in Milli-Q water at concentrations used for the standard MOs alone. The solutions were then heated to 100°C for 10 minutes and then slowly cooled to room temperature to permit hybridization. These solutions were stored as stock solutions at room temperature in the dark. At the appropriate time points, embryos were placed in petri dish and illuminated for 10–15 minutes with a light from a broad spectrum LED light source that passed through a Zeiss AxioZoom. V16 macroscope equipped with a FL Filter Set 49 for DAPI (Thornwood, NY, USA) at a magnification of 15x.

### Mosaic analysis

Mosaic retinas were produced by blastomere transplantation [41]. Clutches of embryos from wild-type matings were dechorionated and injected at the 1-to 4-cell stage with morpholinos and a lineage-tracing label (1∶9 mix of lysine fixable rhodamine-dextran (Molecular Probes) at a total concentration of 5% w/v). At the 1000-cell stage, 10–40 donor cells from morpholino-injected embryos or wild-type embryos were transplanted to the animal pole of the dechorionated wild-type hosts, the region fated for eye and forebrain [42]. Host embryos were fixed in 4% paraformaldehyde at 4.5 dpf. Donor cells in host embryos were assessed by microscopy as described below. At least four independent blastomere transplantation experiments were conducted, with n≥8 surviving embryos from each genotype combination.

### Histology and electron microscopy

Larvae were fixed in a primary fixation solution of 2.5% glutaraldehyde, 1.0% paraformaldehyde prepared in a 0.06 M phosphate rinse buffer for 1.5 hours at 4°C. Larvae were then postfixed in 1% osmium tetroxide (prepared in 0.06 M phosphate rinse buffer for 1.5 hours and then dehydrated through a series of ethanol-water solutions. Specimens were then infiltrated in araldite resin. Fixation and dehydration was facilitated by a Pelco Biowave cold microwave. Transverse serial sections (1.0 µm) were cut and stained with an aqueous solution of 1% methylene blue, 1% azure blue, 1% borax. For electron microscopy transverse sections (0.1 µm) were cut and stained with uranyl acetate and lead citrate and viewed on a JOEL 1200EX transmission electron microscope.

### SDS-PAGE and Western blotting

Adult retinas were homogenized in Laemmli buffer containing protease inhibitor cocktail and centrifuged for two minutes at 1000×g. Two dilutions of the soluble fraction were separated by electrophoresis on 5–15% gradient SDS-PAGE gels (Biorad, Hercules, CA), and transferred to PVDF membranes. Rabbit anti-Pard3 (1∶200) was used for Western blotting using standard protocols.

### Immunohistochemistry and immunocytochemisry

A rabbit polyclonal anti-Pard3 antibody was generated against the synthetic peptide SPYTQKQNGRNGHPSTSDRY, which corresponds to amino (amino acids 1093–1112 in the C-Terminus of zebrafish Pard3, and was affinity purified by Open Biosystems. This antibody was used at 1∶200 dilution.

Immunolabeling procedures followed standard protocols, with the following specifications. Whole mount immunostaining was performed as previously described [43] with an anti-acetylated tubulin antibody at 1∶1000. For immunohistochemistry larvae were fixed in 4% paraformaldehyde in phosphate buffered saline with 0.1% Tween-20 (PBST) for 2–18 hours at 4°C. Specimens were then washed twice in PBST and infiltrated with 30% sucrose (in PBST). Larvae were embedded in Tissue Freezing Medium (Triangle Biomedical Sciences) and frozen. Cryosections (10 µm) were mounted on gelatin-coated glass slides and dried for at least two hours at room temperature. Sections were washed in PBST and treated with block solution (PBST, 5% normal goat serum, 0.5% BSA (w/v), 1% DMSO, 1% Tween-20) prior to immunolabeling using standard procedures. Specimens were imaged with a Zeiss AxioImager microscope fitted with an ApoTome and AxioCam using 63x or 100x Plan-Neo-Fluar objectives and utilizing AxioVision software (Zeiss, Thornwood, NY). Each experiment was performed at least three independent times and at least 10 larvae from each condition were sectioned and stained per experiment.

The following antibodies were used: mouse-anti-ZO-1 (Zymed) at 1∶100; mouse anti-acetylated tubulin (Sigma) at 1∶1000; Zpr-1 monoclonal antibody (Zebrafish International Resource Center, ZIRC, Eugene, OR), 1D1 monoclonal antibody (a gift from Dr. James Fadool, Florida State University [44]); rabbit anti-aPKCζ at 1∶1000 (C-20, sc-216, Santa Cruz Biotechnology); rabbit anti-Ift88 at 1∶5000 [45]; and rabbit anti-Crb at 1∶500 (a gift from Dr. Jarema Malicki, University of Sheffield).

### *In situ* hybridization

Antisense riboprobes corresponding to *southpaw* were synthesized and *in* *situ* hybridizations performed as described [46]. Images were obtained on a Stereo LUMAR stereomicroscope (Zeiss).

### Measurements and statistics

Measurements were performed with AxioVision software (Zeiss) and analyzed using GraphPad Prism5. The inner segment lengths were measured from the most proximal edge of the outer plexiform layer to the most distal edge of Zpr-1 staining. To measure the apical domain, the distance from the outer limited membrane to the distal edge of Zpr-1 staining was quantified. All measurements were taken from the central part of the retina in transverse cryosections sections at or near the optic nerve (n = 8–15 larvae) and analyzed using a 1-way ANOVA and Tukey post-test.

### PKC inhibitor treatments

The myristolated PKCζ pseudosubstrate inhibitor (EMD4Biosciences; 539624) and the non-myristolated form (EMD4Biosciences; 539610) were dissolved in water to a concentration of 1 mM and then diluted to a concentration of 3 µM in embryo media containing 0.5% DMSO [36]. Embryos were dechorionated at 50 hours post fertilization (hpf) and added to wells of a 24-well culture plate containing 500 µL of compound at a density of no more than 8 embryos/well. Treatment at earlier time points resulted in lethality within 2–6 hours. The plates were stored at 28.5°C. At 96 hpf, larvae were collected and processed for immunohistochemistry.

## Results

### Localization of polarity proteins in zebrafish photoreceptors

**Figure 1:**
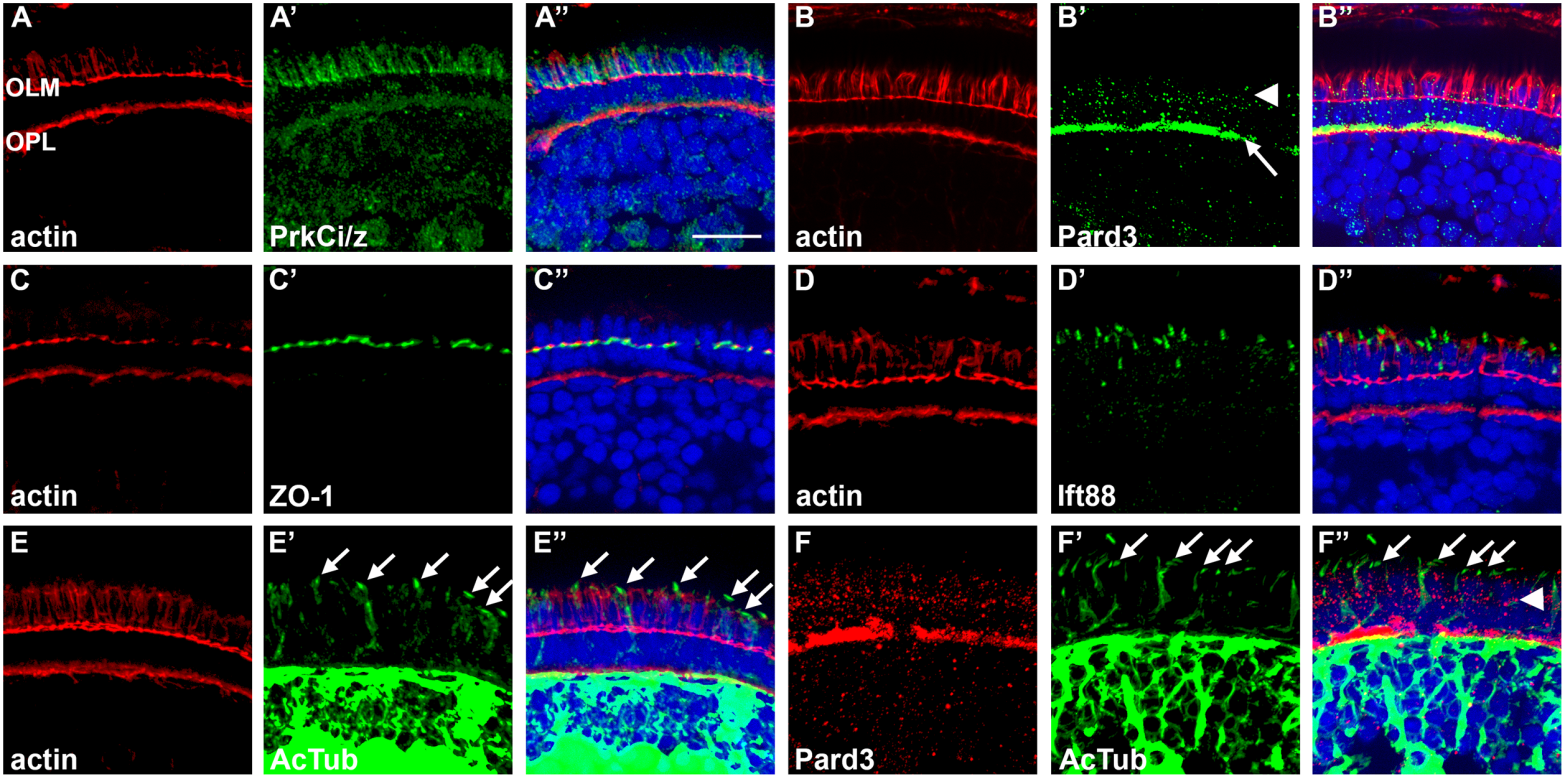
Spatial distribution of cell junction proteins and cilia in 5 dpf zebrafish. (A–E) Left-most panels show transverse cryosections stained with phalloidin (red) to label actin in the adherens junctions at the outer limiting membrane (OLM) and the outer plexiform layer (OPL). The middle panels show staining for determinants of apico-basal polarity (green), while the right-most panels show the merged images. (A1–A3) PrkCi/z immunoreactivity. (B1–B3) Pard3 immunoreactivity localizes to the OPL (arrow) and at cell junctions (arrowhead). (C1–C3) ZO-1 staining at the OLM. (D1–D3) Ift88 immunoreactivity stains cilia. (E1–E3) Acetylated tubulin immunoreactivity was seen in cilia and neural processes throughout the inner nuclear layer and plexiform layers. (F1–F3) Pard3 staining (red) was seen at the OLM (arrowhead), but did not colocalize with acetylated tubulin signal in the cilia (arrows). All sections were counterstained with DAPI (blue). Scale bar = 10 µm.

To investigate the link between polarity proteins and ciliogenesis *in* *vivo*, we first examined the spatial distribution of Pard3 and PrkC in cryosections of larval zebrafish retinas. Previous studies found that Pard3 localized to the apical surface of the retina and brain neuroepithelia at 33 hpf [17]. We generated a rabbit polyclonal antibody against a peptide of Pard3 (see Materials and Methods) and confirmed the specificity of this antibody by Western blotting (Fig. S1). Although the antibody should recognize both splice isoforms, we only detected the 180 kDa protein on Western blots (Fig. S1). Photoreceptor outer segments, which are considered specialized sensory cilia, are well-developed by 5 dpf and we examined the localization of Pard3 and other junction markers relative to cilia proteins (Fig. 1). Phalloidin was used to stain the actin belt at the adherens junctions of the outer limiting membrane (OLM). As previously observed [28], actin localized vertically along the sides of the photoreceptors, immediately apical to the OLM, in a pattern resembling longitudinal fibers within the ellipsoid [47]. As expected, immunostaining for the cell junction proteins protein kinase C iota/zeta (PrkCi), Pard3, and ZO-1 revealed colocalization with actin at the OLM, although each protein had a distinctive spatial distribution (Fig. 1A–C). An antibody that recognizes an epitope shared by PrkCi and the close paralog, protein kinase C zeta (PrkCz), colocalized with actin at the OLM and along the sides of the photoreceptor in the domain apical to the OLM. Crumbs proteins and the FERM domain protein Mosaic eyes (Moe), which function as apical determinants, also localized on the apical side of the OLM [28], [48]. Consistent with earlier studies [17], the strongest Pard3 staining colocalized with actin at the outer plexiform layer (Fig. 1B). Pard3 also localized in a punctate pattern near the OLM, although the signal intensity was less pronounced than at the outer plexiform layer. ZO-1 distribution was restricted to the OLM (Fig. 1C). Immunoreactivity to Ift88, a component of the IFT particle, was seen in connecting cilia as previously described, but did not colocalize with actin (Fig. 1D; [45]). Ciliary axonemes were visualized by immunostaining for acetylated tubulin, which also did not colocalize with actin (Fig. 1D, E). As Pard3 localized to primary cilia in MDCK cells [34], [35], we then asked if Pard3 localized to the photoreceptor connecting cilia or outer segments. While Pard3 was seen with other apical determinant proteins at the OLM, we did not observe Pard3 localizing with acetylated tubulin in the connecting cilia of photoreceptors (Fig. 1F). These results show that while Pard3, PrkC, and Crumbs proteins appeared at cell junctions, Pard3 was found at the outer plexiform layer, PrkC and Crumbs were seen in the domain immediately apical to the OLM, and none could be detected in the cilia *in* *vivo*.

### Genetic mosaic analysis of *pard3*-deficient cells

Pard3 physically interacts with PrkCi and this interaction is necessary for establishing apical identity cultured cells [23]. Testing the role of Pard3 or PrkCi in photoreceptors, however, poses a number of experimental challenges. Complete loss-of-function mutations in genes required for apico-basal polarity, including *heart and soul* (*has*/PrkCi), *mosaic eyes* (*moe*), the Crumbs ortholog *oko meduzy* (*ome*), and *nagie oko* (*nok*/Stardust), disrupt the laminar organization of the retina and impair photoreceptor cell differentiation [28], [38], [39], [49]–[51]. Additionally, PrkCi and PrkCz function redundantly and suppression of both proteins may be necessary to fully test the role of PrkC activity in photoreceptors. These phenotypes are, however, cell-non-autonomous and can be rescued in genetically mosaic animals [38], [49], [50]. Injection of 4–5 ng of a previously characterized *pard3* morpholino (MO1) fully suppressed Pard3 function but also caused cyclopia and disrupted retinal lamination and patterning [15], [17]. In an attempt to examine the role of Pard3 on photoreceptor structure, morpholino oligonucleotides (MOs) against *pard3* were injected into 1-cell embryos and we conducted genetic mosaic experiments using rhodamine-dextran as a lineage dye. At 4 dpf, wild-type donor cells contributed to all retinal cell types in wild-type hosts (Fig. S2A). The *pard3* morphant donor cells also migrated to the appropriate laminar position in the wild-type hosts, indicating that the retinal cell positioning defect is cell-non-autonomous. We noticed, however, that the *pard3* morphant cells formed few, if any, photoreceptors and only individual photoreceptors were ever observed in mosaic retinas (Fig. S2B). These rare *pard3*-deficient photoreceptors formed normal adherens junctions with neighboring wild-type cells, as determined by actin and ZO-1 staining (data not shown). In agreement with previous results [38], we also noticed that few photoreceptors formed when cells from *prkci* morphants or *prkci/prkcz* double morphants were transplanted into wild-type hosts (Figs. S2C and S2D). These results suggest that Pard3 and PrkC may function to limit cell fate or differentiation. Indeed, the loss of Pard3 activity in progenitor cells within the subventricular zone of the mouse cerebral cortex resulted in early neurogenic differentiation and fewer progenitor cell divisions [52]. This may explain why *pard3-*deficient cells largely failed to differentiate. Given these results, we were reluctant to draw conclusions regarding cilia formation from single *pard3*-deficient photoreceptors due to the earlier effect of *pard3* loss on retinal progenitor cell proliferation/differentiation.

### Partial suppression of *pard3* by morpholinos

**Figure 2:**
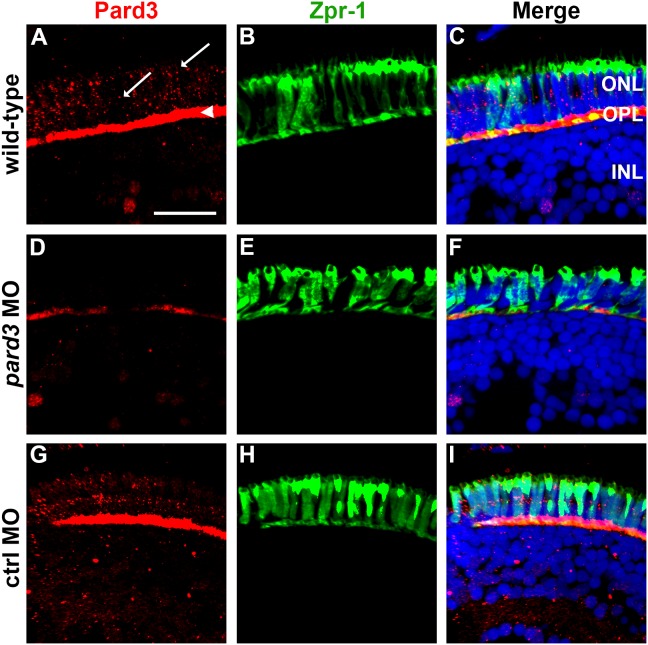
Pard3 expression is reduced at 5 dpf following injection of *pard3* morpholinos. (A–C) Transverse cryosections of wild-type retinas, (D–F) *pard3* morphant retinas and (G–I) control morphant retinas with Pard3 antiserum (left column) and the monoclonal antibody zpr-1 (middle column). The right panels show the merged images. Pard3 immunoreactivity was seen in the outer plexiform layer (OPL) and at cell junctions (A, arrowhead and arrows, respectively) in wild-type and control MO retinas but was significantly reduced in morphants retinas. All sections were also counterstained with DAPI (blue). ONL = outer nuclear layer; OPL = outer plexiform layer; INL = inner nuclear layer. Scale bar = 10 µm.

To avoid the effects on retinal patterning and photoreceptor differentiation, but still partially suppress Pard3, doses of MO1 ranging from 1.2–1.5 ng were injected into 1-cell embryos. To verify that Pard3 expression was reduced, cryosections of wild-type and *pard3* MO1-injected larvae were immunolabled with Pard3 antisera and colabeled with zpr-1, a marker for red-green double cones (Fig. 2). Pard3 immunostaining was seen at the OPL (Fig. 2A, arrowhead) and in two rows of cell junctions (Fig. 2A, arrows). In morphant retinas, Pard3 immunoreactivity was significantly reduced at the OPL and almost entirely absent at the OLM (Fig. 2D). Importantly, the Zpr-1 staining showed that photoreceptor differentiation was not impeded following Pard3 suppression (Fig. 2F). Injection of a control morpholino, which contained a 6-basepair mismatch, did not affect Pard3 expression (Fig. 2G–I). From these results, we concluded that smaller doses of *pard3* morpholino could reduce Pard3 proteins levels without impairing photoreceptor differentiation.

**Figure 3:**
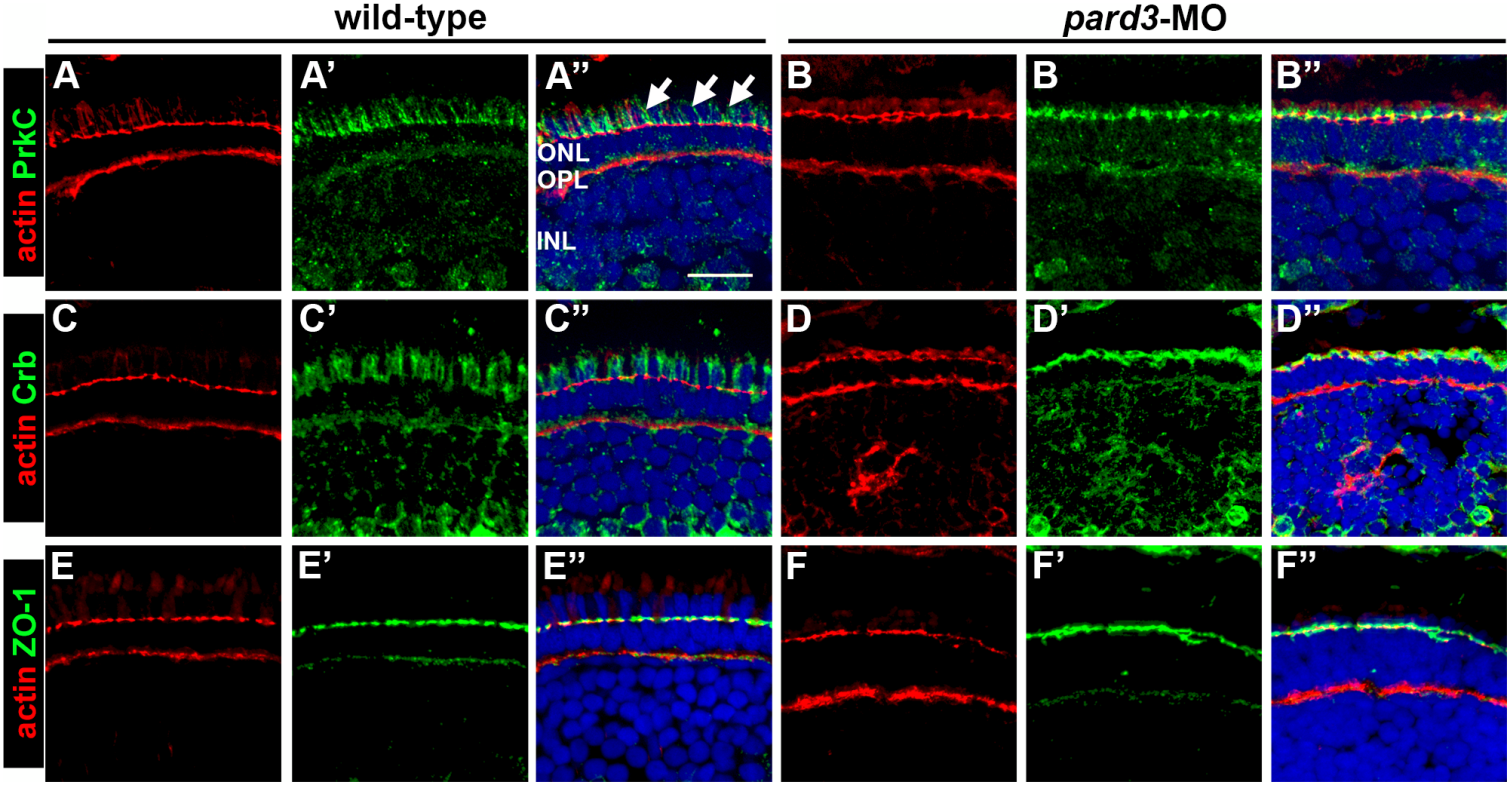
Effects of *pard3* suppression on apical determinants. Immunohistochemistry was performed on transverse cryosections of wild-type and *pard3* morphants. Phalloidin was used to label actin (red) in all panels. (A–B) Transverse cryosections of wild-type and *pard3* morphants stained with phalloidin (red) and PrkCi immunoreactivity (green). Arrows mark longitudinal fibers in the ellipsoid. (C–D) Cryosections stained with phalloidin (red) and Crb immunoreactivity (green). (E–F) Cryosections stained with phalloidin (red) and ZO-1 immunoreactivity (green). Sections were also counterstained with DAPI (blue). ONL = outer nuclear layer; OPL = outer plexiform layer; INL = inner nuclear layer. Scale bar = 10 µm.

The formation of cellular junctions requires Pard3 and suppression of Pard3 by RNAi disrupted the localization of other polarity proteins to tight junctions in cultured cells [20]. Studies in zebrafish, however, found normal ZO-1 localization to the apical midline in 12–13 somite *pard3* morphants [15]. We asked if the loss of Pard3 at the OLM affected the cellular distribution of other polarity proteins within photoreceptors. Morpholino knockdown of *pard3* did not alter the OLM localization of actin but the staining pattern appeared rough and less organized (compare Fig. 3A to 3B, D, F). The localization of PrkC to the apical side of the OLM was unaffected in *pard3* morphants, but the apical surface (Fig. 3B”; arrows) was reduced and most of the signal was aggregated at the OLM (Fig. 3B). Immunohistochemistry with a polyclonal antibody that recognizes multiple Crumbs polypeptides gave a staining pattern similar to that of PrkC (Fig. 3C). In *pard3* morphants, Crumbs staining was aggregated at the OLM and the apical membrane was reduced (Fig. 3D). ZO-1 staining was not lost in *pard3* morphants, although the signal at the OLM was less discrete (compare Figs. 3E and F). Taken together, these results suggest that partial suppression of Pard3 was not sufficient to block the normal localization of polarity proteins or the formation of adherens junctions.

**Figure 4:**
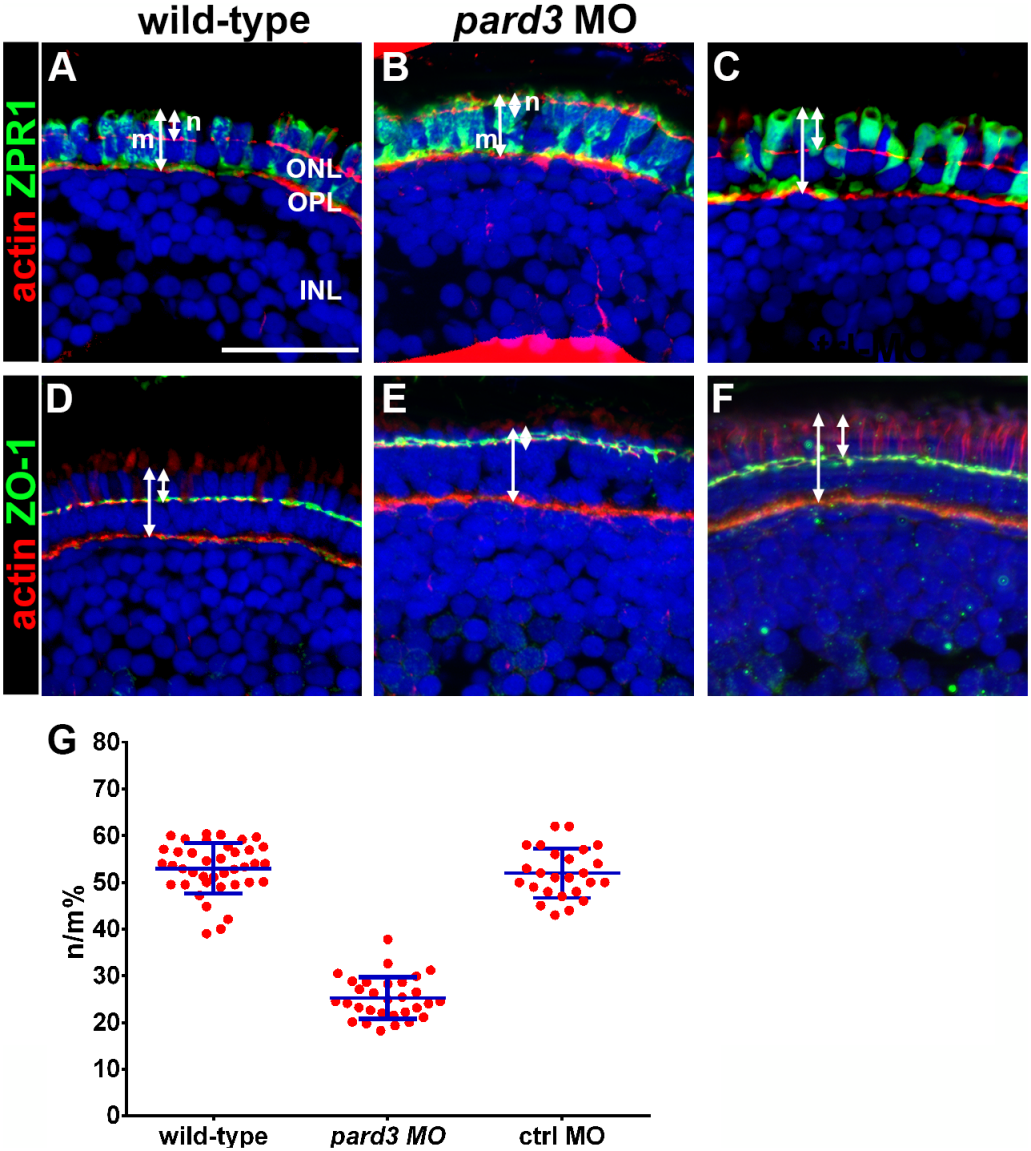
Apical domain size is reduced in *pard3* morphants. (A–C) Transverse cryosections of 5 dpf retinas were stained with phalloidin to label actin (red) and zpr-1 to label double cones (green). The length of the photoreceptor (m) and the length of the apical region of the inner segment (n) are noted by arrows. (D–F) Transverse sections stained with phalloidin (red) and ZO-1 (green) to label the outer limiting membrane. Sections were also counterstained with DAPI (blue). (G) Quantification of apical domain as a percentage of total photoreceptor inner segment length. All values are plotted as a percentage of n/m, as defined in the text. Wild type = 53%; *pard3* morphants = 25%; control morphants = 52%. Blue error bars indicate the mean ± standard deviation. ONL = outer nuclear layer; OPL = outer plexiform layer; INL = inner nuclear layer. Scale bar = 20 µm.

Pard3 promotes formation of the apical membrane domain [23], and the loss of PrkCi and Crumbs from the region immediately apical to the OLM may reflect a reduction in the apical domain size. The OLM divides the photoreceptor membrane into apical and basolateral domains, with the inner segment corresponding to the area between the OLM and the base of the outer segment. When wild-type retinas are stained with Zpr-1 at 5 dpf, the inner segments of cone photoreceptors are partitioned almost equally between the apical and basolateral domains [28]. We stained photoreceptors with Zpr-1 and quantified the size of the apical inner segment domain (Fig. 4A; “n”) relative to total length of Zpr-1 staining (Fig. 4A; “m”). Consistent with previous reports [28], the apical domain accounts for approximately 50% of the length of Zpr-1 staining in 5 dpf larvae (Fig. 4A, quantified in Fig. 4G). In *pard3* morphants, the apical domain was considerably smaller and was less than 30% of the total length of Zpr-1 staining (Fig. 4B). We found similar results when we labeled the OLM with ZO-1 and measured the distance from the outer plexiform layer to the most distal point of DAPI staining, which corresponded to the nuclei of the red/green double cones (Fig. 4D). Control morpholinos had no effect on apical domain size (Fig. 4C, F). Overall, these results indicate that low doses of *pard3* morpholino reduced the apical domain in photoreceptors.

### Photoreceptor outer segment formation is reduced in *pard3* morphants

**Figure 5:**
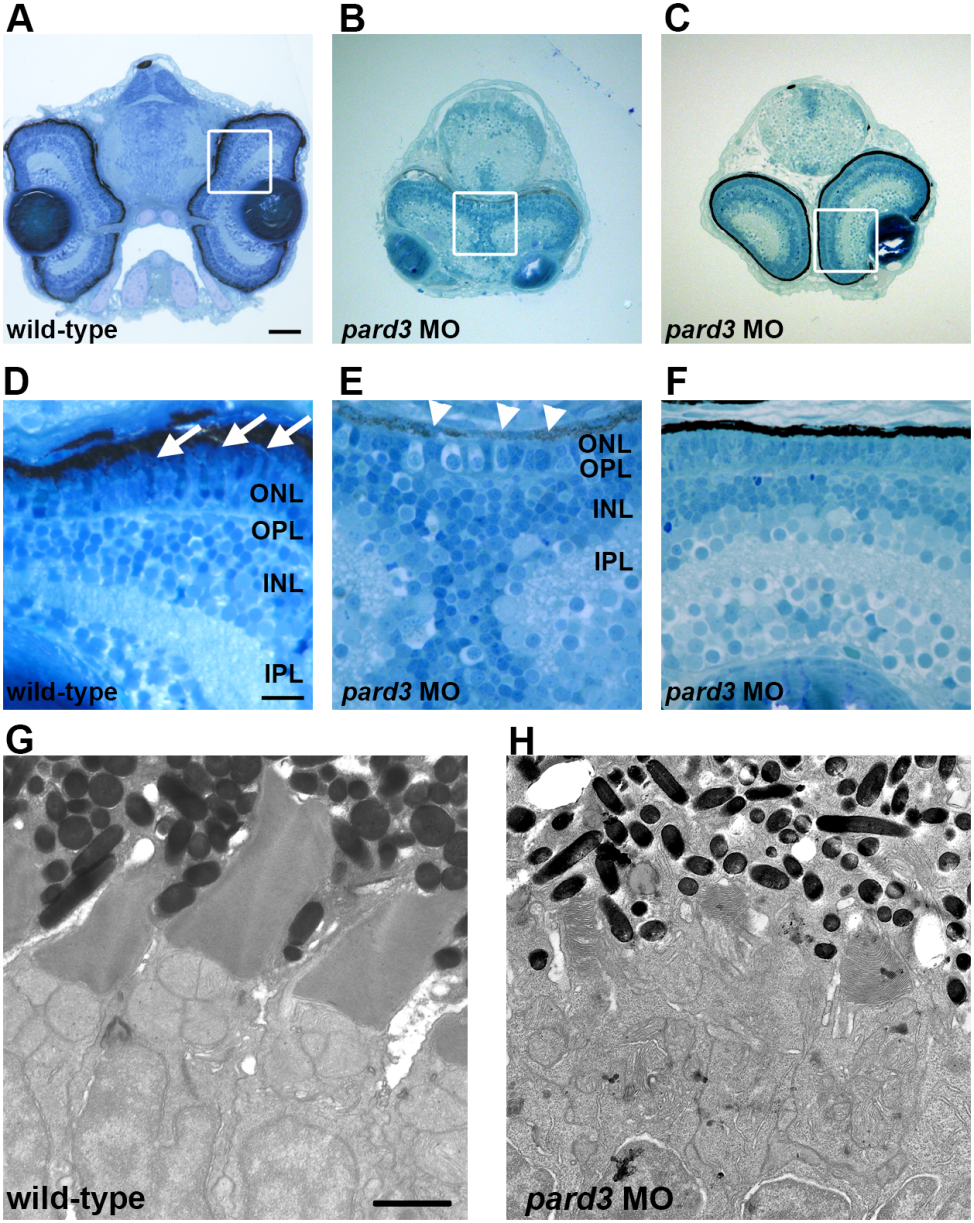
Morpholino suppression of Pard3 causes variable cyclopia and loss of photoreceptor outer segments. (A–C) Transverse plastic sections of 4 dpf larvae heads from wild-type and *pard3* morphants show variable degrees of cyclopia but normal lamination. (D–F) Higher magnification of the boxed areas in A–C revealed the photoreceptor outer segments in wild-type retinas (Panel D, arrows), which were missing in *pard3* morphants. Morphants with fused eyes also exhibited patchy pigmentation in the RPE (Panel E, arrowheads). (G–H) Transmission electron microscopy of wild-type and *pard3* morphant retinas showed truncation of photoreceptor outer segments. ONL, outer nuclear layer; OPL, outer plexiform layer; INL, inner nuclear layer; IPL, inner plexiform layer. Scale bar = 50 µm (A–C); 10 µm (D–F); 1 µm (G–H).

As cilia formation occurs on the apical surface of the inner segment, we next asked photoreceptor outer segment formation was affected. Histological sections of 4 dpf larvae confirmed that retinal lamination and organization was largely unaffected by suppression of Pard3 (Fig. 5), although some degree of disorganization was occasionally observed at higher morpholino concentrations (data not shown). Even at lower doses of morpholino, however, the *pard3* morphants exhibited varying degrees of cyclopia, with some larvae having partially fused eyes and other larvae exhibiting only a slight reduction in distance between the eye fields (Fig. 5A–C). Morphants with more pronounced cyclopia also showed reduced pigmentation of the RPE when compared to morphants without fused eyes (Fig. 5, panels E and F, respectively). Photoreceptors were present in the outer nuclear layer of *pard3* morphants, but outer segments were not observed (Fig. 5D–F). Transmission electron microscopy confirmed that outer segments were significantly reduced, and often missing, from photoreceptors in *pard3* morphants (Fig. 5G–H). The truncated outer segments that did form on *pard3* morphant photoreceptors contained regularly stacked disc membranes, indicating that loss of Pard3 does not affect disc membrane organization. The positioning of the mitochondria and nucleus were normal, suggesting that apicobasal positioning of organelles was not disrupted.

**Figure 6:**
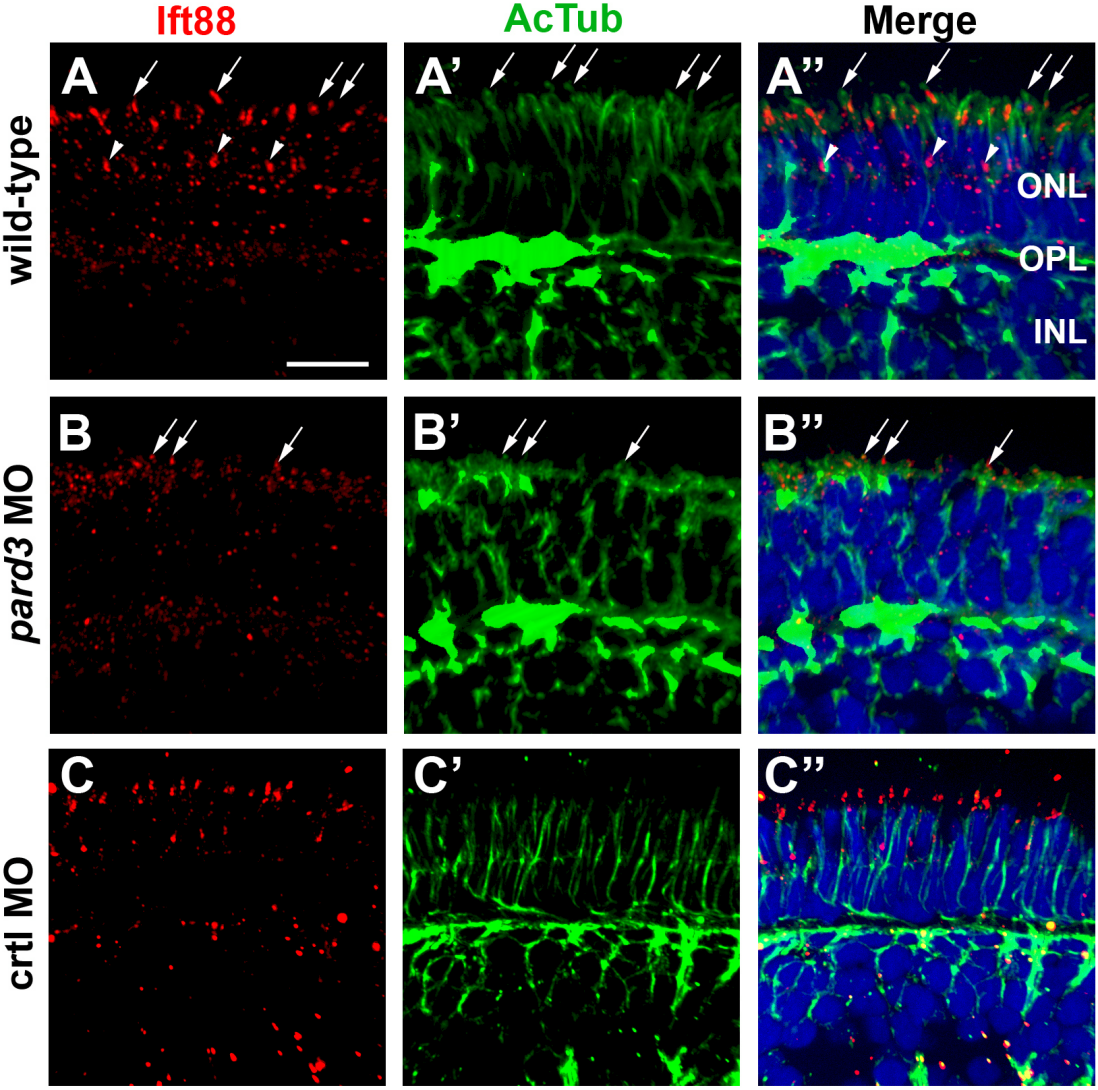
Connecting cilia are reduced in *pard3* morphants. (A–C) Transverse cryosections were stained for Ift88 (left, red) and acetylated tubulin (middle, green) to label cilia in 5 dpf retinas. Merged images are shown in right panels. Ift88 localizes to the connecting cilia emanating from the apical surface of photoreceptors (arrows). Staining is also seen in connecting cilia of UV cones (arrowheads), which are tiered below the other cones. Sections were also counterstained with DAPI (blue). ONL = outer nuclear layer; OPL = outer plexiform layer; INL = inner nuclear layer. Scale bar = 10 µm.

The formation and growth of photoreceptor outer segments requires the process of Intraflagellar Transport, or IFT [45], [53], [54], and the Par-PrkCi complex has been linked to IFT [34]. Pard3 physically associates with Kif3a, the anterograde motor for IFT, and PrkCi binds to the complex through its association with Pard3 [34], [55]. Furthermore, the interaction between the Par-PrkCi complex and the Crb complex is required for photoreceptor differentiation in Drosophila [26]. The absence of outer segments in *pard3* morphants may reflect a failure in apical assembly of IFT particles. To investigate whether loss of Pard3 affected the localization of ciliary proteins, we examined transverse sections stained with antibodies against acetylated tubulin to label cilia and with Ift88, a component of the IFT particle. Although acetylated tubulin staining was concentrated at the apical surface of *pard3* morphants, very few cilia could be seen projecting toward the RPE (Fig. 6, compare panels A” and B”). Ift88 immunoreactivity was also reduced in *pard3* morphants, although the remaining signal localized at the apical surface (Fig. 6B and B”).

**Figure 7:**
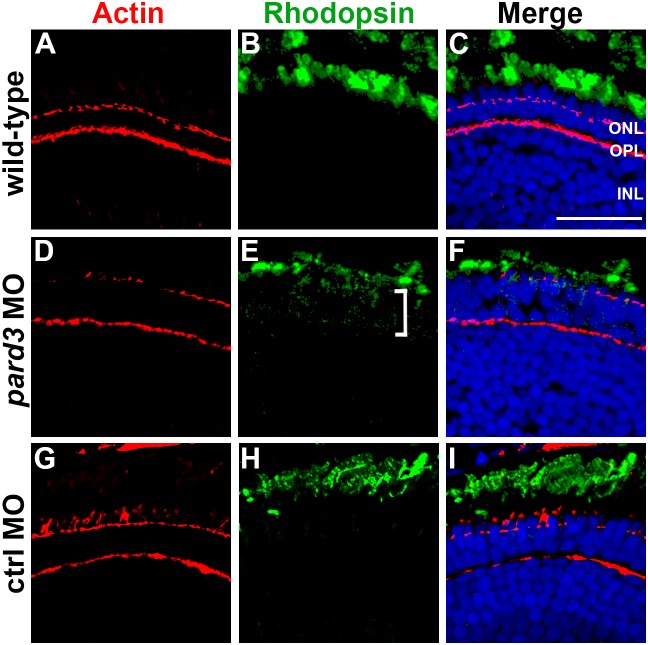
Rhodopsin is mislocalized in *pard3* morphants. (A) Immunohistochemistry was performed on transverse cryosections through the retina of 5 dpf wild-type larvae. Left most panels show phalloidin staining to label actin (red). Rhodopsin immunoreactivity (green) is shown in middle panels. (D–F) In *pard3* morphants, outer segments appear slightly shorter and opsin staining within the inner segment is observed (bracket). (G–I) Rhodopsin localizes normally in control morphants. Sections were also counterstained with DAPI (blue). ONL = outer nuclear layer; OPL = outer plexiform layer; INL = inner nuclear layer. Scale bar = 20 µm.

IFT facilitates the trafficking of proteins, such as rhodopsin, to the photoreceptor outer segment. While opsin molecules function primarily to detect photons of light for the visual response, they are also the most abundant proteins in the outer segment and provide structural support to the outer segment [56]. Mutations in components of the IFT particle or in Kif3a cause opsin to accumulate in the photoreceptor inner segment and even partial loss of IFT results in severe outer segment defects [45], [53], [54], [57]. As *pard3* morphants show reduced levels of Ift88 and loss of outer segments, we anticipated that rhodopsin would be mislocalized. Indeed, opsin immunoreactivity could be seen in the inner segments of *pard3* morphants (Fig. 7E, bracket) and the outer segment staining was reduced (Fig. 7E, compare to 7B). Thus, the loss of Pard3 reduces outer segment size and results in mislocalization of opsin.

### Temporal regulation of *pard3* and *prkc* by photo-morpholinos

In an attempt to more effectively block Pard3 and PrkC activity at later time points, we utilized sense photo-active morpholinos (S-photo-MOs) to keep morpholinos temporarily inactive (See Materials and Methods and [40]). Standard MOs and the complementary S-photo-MOs specific for *pard3*, *prkci* or *prkcz* were hybridized to form dimers *in* *vitro* prior to injection into 1-cell embryos. Larvae injected with MO/S-photo-MO hybrids specific for either *pard3* or *prkci* did not exhibit any overt morphological phenotypes or disruption to retinal structure in the absence of UV irradiation (Figs. S3 and S4). Larvae injected with the MO/S-photo-MO hybrids and subsequently irradiated with UV at 5 hpf showed both morphological and retinal lamination phenotypes that resembled the phenotypes seen in *pard3* morphants and *prkci* mutants (Figs. S3 and S4; [17], [38], [51]). No adverse phenotypes were observed in larvae injected of S-photo-MOs alone. These results suggest that embryos retain endogenous Pard3 or PrkCi activity until the S-photo-MO is cleaved by UV light and the unmodified MO can target the cognate mRNA to suppress function.

**Figure 8:**
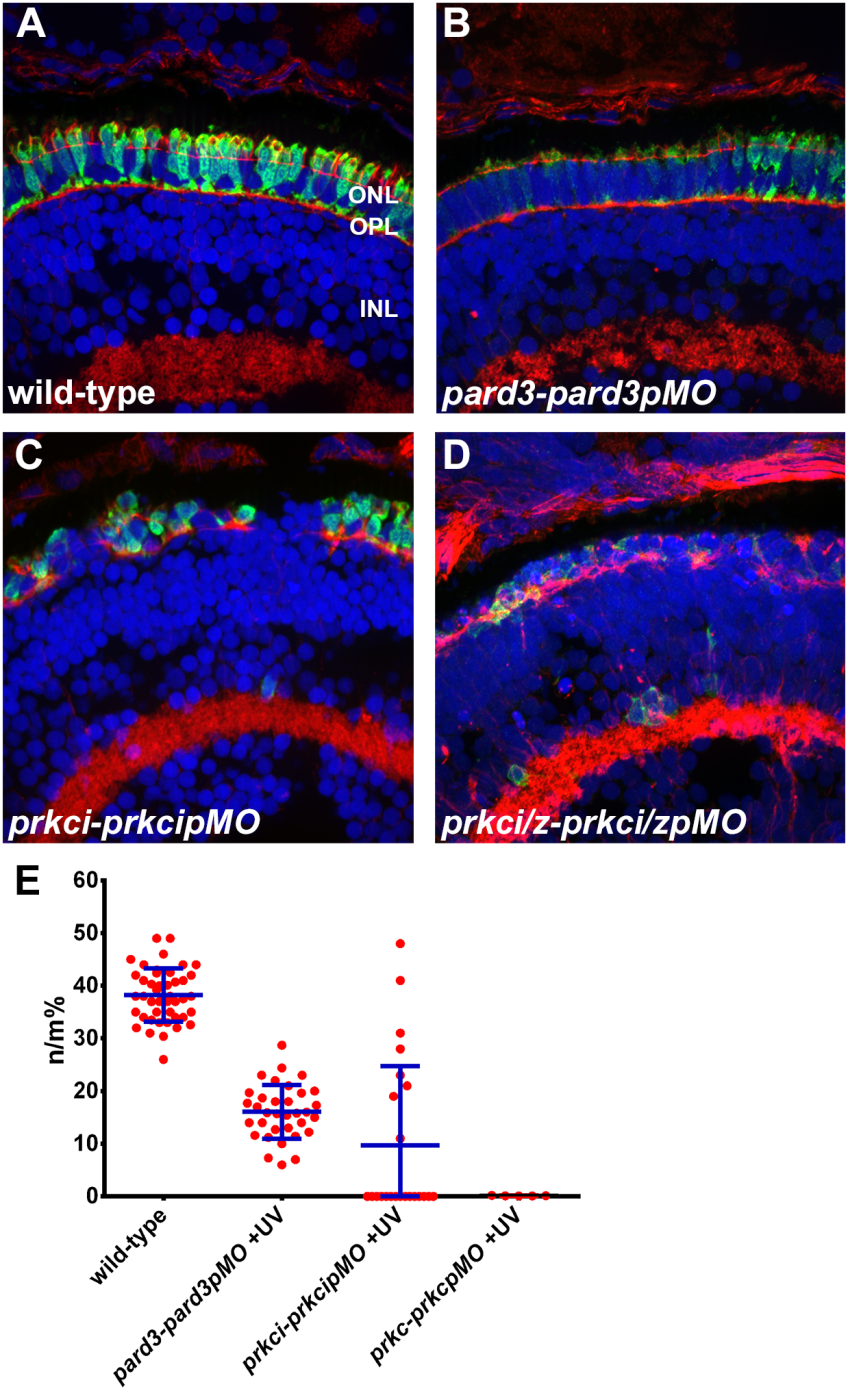
Photo-Morpholinos reduce apical domain size. (A–D) Transverse cryosections of 4 dpf retinas were stained with phalloidin to label actin (red) and zpr-1 to label double cones (green). Sections were also counterstained with DAPI (blue). (E) Quantification of apical domain as a percentage of total photoreceptor inner segment length. All values are plotted as a percentage of n/m, as defined in the text. Wild type = 38%; *pard3-pard3pMO* morphants = 16%; *prkci-prkcipMO* morphants = 9.72%; *prkc-prkcpMO* was non-detectable. Blue error bars indicate the mean ± standard deviation. ONL = outer nuclear layer; OPL = outer plexiform layer; INL = inner nuclear layer. Scale bar = 20 µm.

**Figure 9:**
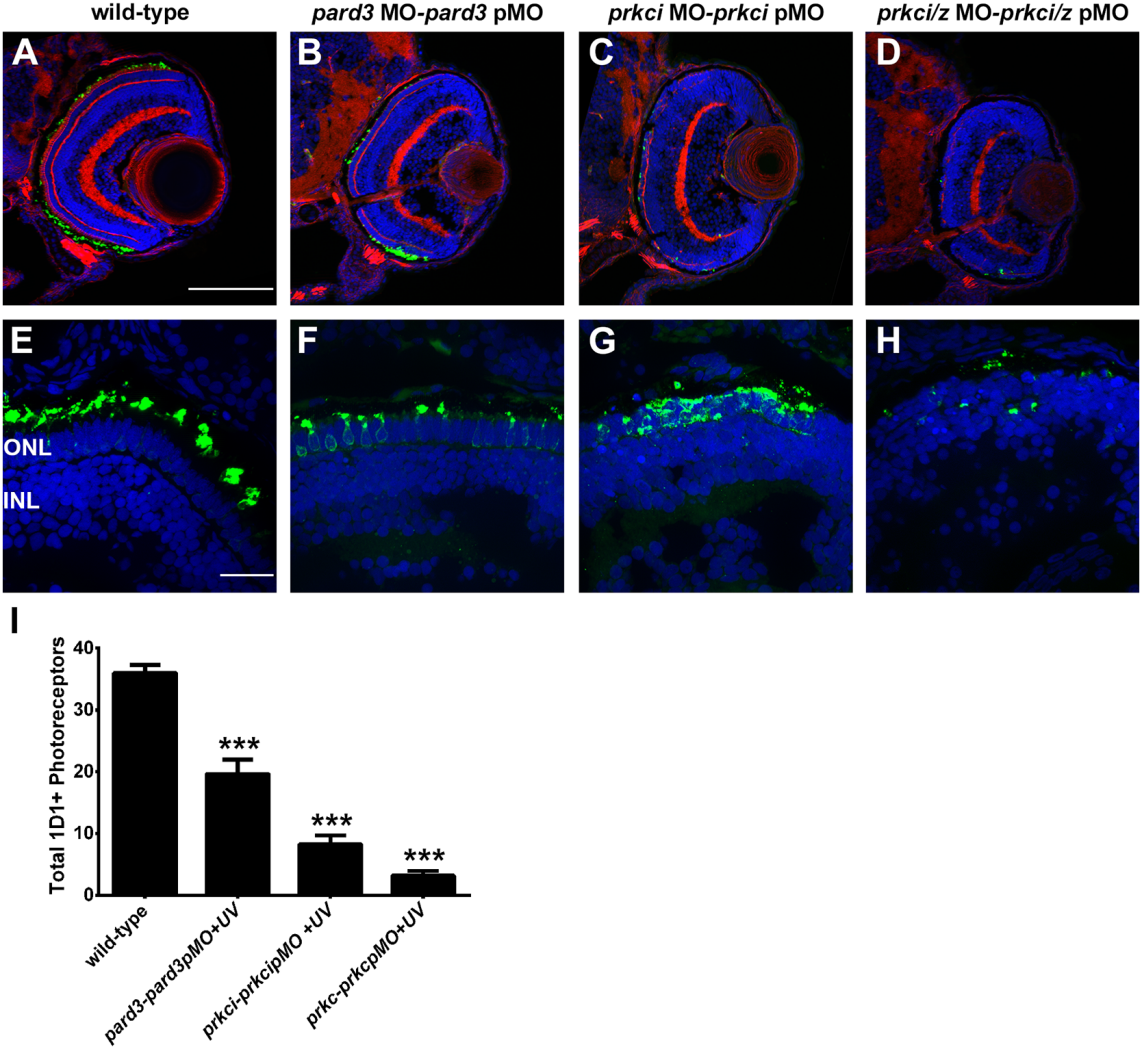
Photo-Morpholinos cause rhodopsin mislocalization and loss of rod photoreceptors. (A, E) Transverse retinal cryosections from 4 dpf wild-type; (B, F) *pard3*MO-*pard3*pMO; (C, G) *prkci*MO-*prkci*pMO; (D, H) *prkci/z*MO-*prkci/z*pMO injected larvae. Embryos were injected with MO/S-photo-MO hybrids and irradiated at 30 hpf with UV light. Immunohistochemistry was performed on transverse cryosections of 4 dpf larvae and stained with 1D1 to label rhodopsin (green) and phalloidin (red) and counterstained with DAPI to label nuclei. Bottom row shows 1D1 and DAPI staining only. (I) Quantification of total 1D1+ rod photoreceptors in 10 micron cryosections. (*** = p≤0.0001) ONL = outer nuclear layer; INL = inner nuclear layer. Scale bar = 100 µm (A–D) or 20 µm (E–H).

To block the activity of Pard3 or PrkC at time points corresponding to the beginning of photoreceptor ciliogenesis, embryos were injected with MO/S-photo-MO hybrids and irradiated with UV light at 30 hpf. Although photoreceptor differentiation begins at 52 hpf, we found that irradiation at 30 hpf was required to give robust photoreceptor phenotypes at 4 dpf consistent with the loss of Pard3/PrkC activity. This may reflect the time needed to for the S-photo-MO to fully degrade, the MO to bind and inhibit the cognate mRNA, and for protein turnover. Irradiation at later times (e.g. 42 hpf or 50 hpf) resulted in little to no phenotype at 4 dpf (data not shown). Similar to the previous experiments, morphant retinas were stained with Zpr-1 and we quantified the size of the apical domain. At 4 dpf, the apical domain constitutes approximately 40% of the wild-type photoreceptors (Figs. 8A, E). When larvae were UV irradiated at 30 hpf, the apical domain was significantly reduced compared to wild-type larvae (Fig. 8; n/m value: 38% vs. 16%, p<0.0001; n = ≥34). When larvae were irradiated at 30 hpf following co-injection of MO/S-photo-MO hybrids simultaneously targeting *prkci* and *prkcz*, the apical domain was essentially lost (Fig. 8D). The outer limiting membrane could not be detected by phalloidin, and zpr-1 staining was lost, suggesting that cells in the outer nuclear layer failed to differentiate into photoreceptors. Larvae injected with MO/S-photo-MO hybrids also showed significant reduction in the number of 1D1+ rod photoreceptors and mislocalization of rhodopsin (Fig. 9). Whereas rhodopsin localized predominantly to the outer segment of wild-type photoreceptors, suppression of Pard3 resulted in small outer segments and inner segment localization of rhodopsin. Suppressing PrkCi alone or all PrkC activity had more pronounced phenotypes, with few rhodopsin-positive cells being observed and rhodopsin localizing throughout the inner segments and cell bodies (Fig. 9). We quantified the number of 1D1+ rod photoreceptors in 10-micron cryosections. Compared to wild-type larvae, the number of rods was reduced 45% in *pard3* photo-morphants (36 vs. 20 per section; p≤0.001; n ≥10). Rods were reduced by 77% in *prkci* photo morphants and 92% following in larvae injected with both *prcki* and *prkcz* photo-MOs (Fig. 9I). As photoreceptors were missing almost entirely from the *prcki/prkcz* photo-morphants, cilia lengths were not assessed.

### Pharmacological inhibition of PrkC reduced apical membrane size

**Figure 10:**
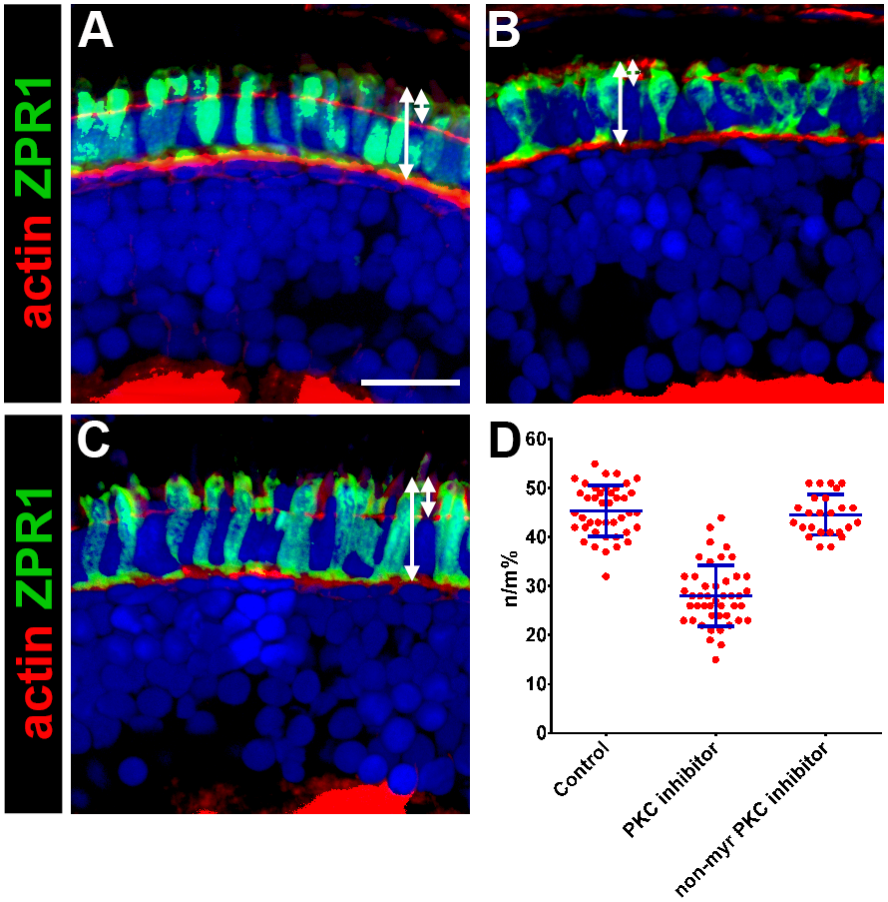
Pharmacological inhibition of PrkC activity reduces apical domain size. (A–C) Transverse cryosections of 4 dpf retinas were stained with phalloidin to label actin (red) and zpr-1 to label double cones (green). (A) DMSO-control treated larvae; (B) Larvae treated with 3 µM PrkC inhibitor; (C) Larvae treated with 3 µM control inhibitor. Sections were also counterstained with DAPI (blue). (D) Quantification of apical domain as a percentage of total photoreceptor inner segment length. All values are plotted as a percentage of n/m, as defined in the text. Control = 45%; PrkC inhibitor = 28%; non-myristoylated control inhibitor = 44%. Blue error bars indicate the mean ± standard deviation. Scale bar = 10 µm.

**Figure 11:**
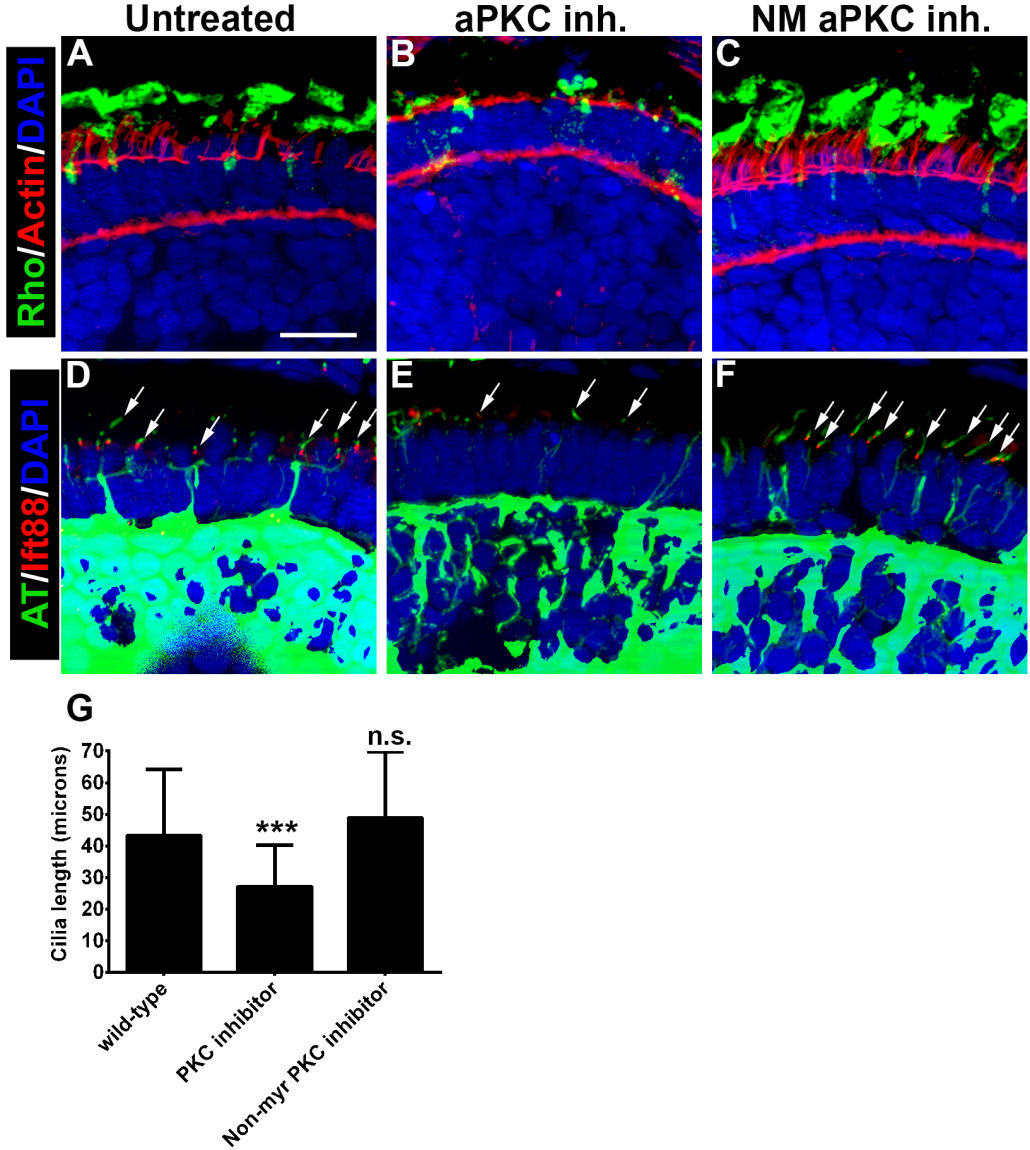
Pharmacological inhibition of PrkC activity reduces cilia length and causes opsin mislocalization. (A–C) Transverse cryosections of 4 dpf retinas were stained with phalloidin to label actin (red) and 1D1 to label rhodopsin (green). (D–F) Immunohistochemistry on cryosections to label Ift88 in cilia (red) and acetylated tubulin to label ciliary microtubules (green). Arrows identify cilia. Sections were also counterstained with DAPI (blue). (G) Quantification of cilia length. Cilia length in 4 dpf wild-type photoreceptors (43.2 microns) and the non-myristoylated control inhibitor (48.9 µm) were not statistically different from each other, but both were statistically different from larvae treated with the PrkC inhibitor (27.1 microns). Error bars show standard deviation. (***p≤0.0001) Scale bar = 10 µm.

Finally, we used a pharmacological approach to test the role of PrkC activity in photoreceptors. Zebrafish embryos were treated with a cell-permeable PKC-specific inhibitor peptide (3 µM, myristoylated PKCζ pseudosubstrate inhibitor), which inhibits the activity of both PrkCi and PrkCz, and can block ciliogenesis in MDCK cells [34], [58]. As a control, we used a non-myristoylated form of the peptide. Zebrafish were treated with the PKC inhibitor at 50 hpf, a time at which photoreceptors have become post-mitotic but have not yet initiated outer segment formation [59], and were examined at 4 dpf, after two days of exposure to the peptide. Treatment at 24 hpf or 36 hpf caused lethality within 2–6 hours and embryos effectively disintegrated, possibly due to disrupting the integrity of the ectodermal epithelia. Similar to the previous experiments, retinas were stained with Zpr-1 and we quantified the size of the apical domain (Fig. 10). Compared to wild-type, inhibition of PrkC significantly reduced the size of the apical domain (45% vs. 28%; p≤0.001; n≥12 larvae). Treatment with the non-myristolated form of the inhibitor did not have any effect on apical domain size (Fig. 10C, D). Rhodopsin was mislocalized in animals treated with the PKC inhibitor but not in control animals (Figs. 11A–C). Cilia length was also reduced following inhibition of PrkC (Fig. 11D–G) but the number of cilia was not statistically altered (data not shown).

### Loss of *pard3* or *prkc* affected cilia in Kupffer’s Vesicle

**Figure 12:**
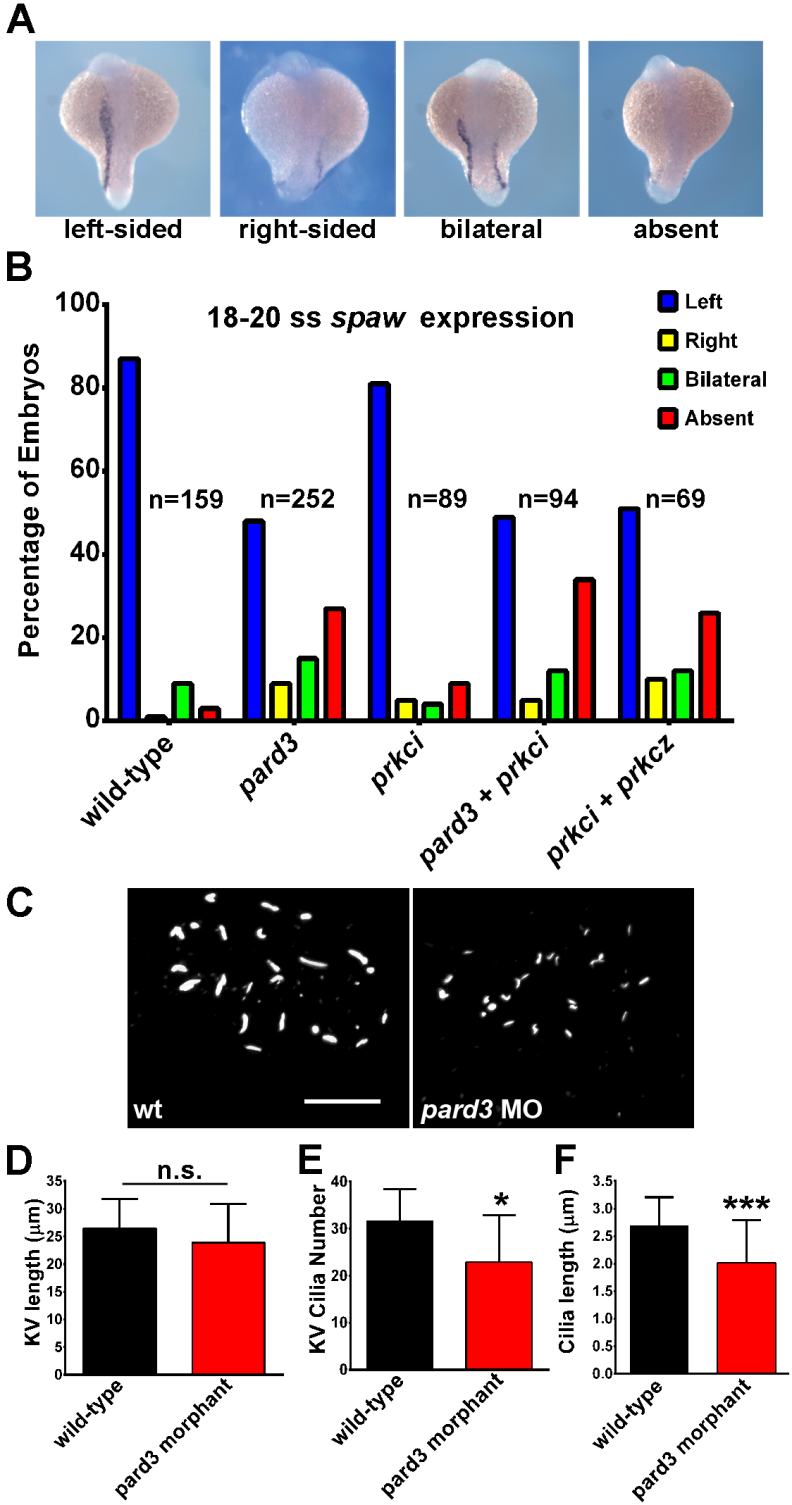
Left-right asymmetry requires *pard3*. (A) In situ hybridization for *southpaw* (*spaw*) expression in the lateral plate mesoderm in 18–20 somite stage embryos. *pard3* morphants show left-sided, right-sided, bilateral, or an absence of *spaw* expression. (B) Graphical summary from three independent experiments showing the percentage of control and morphant embryos with left-sided (L), right-sided (R), bilateral (B), or absence (A) *spaw* expression. The number (n) of embryos analyzed is given next to each set of bars. (C) KV cilia in a wild-type and *pard3* morphant. (D) Quantification of KV length across the longest axis. (E) Quantification of KV cilia number. (F) Quantification of KV cilia length. Bars show mean ± standard deviation (*p≤0.05; ***p≤0.0001) Scale bar = 20 µm.

Vertebrates exhibit an asymmetry across the left-right (LR) axis that is linked to the function of motile cilia in Kupffer’s Vesicle (KV) in zebrafish, or the node of mouse (reviewed in [60]. Disruption of KV specification or of KV cilia function randomizes LR asymmetry in zebrafish [61]–[63]. The *nodal-*related gene *southpaw* (*spaw*) is one of the earliest markers of LR asymmetry and is normally expressed in the left lateral plate mesoderm of 18–20 somite stage embryos [64]. Approximately 87% of wild-type embryos express *spaw* on the left side (L) with small percentages (<9%) showing right-sided (R) expression, bilateral (B) expression, or an absence (A) of expression (Fig. 12A). Even with modest suppression of *pard3* (1.2 ng morpholino), 48% show left-sided expression of *spaw*, while the others show right-sided (9%), bilateral (15%), or an absence (27%) of *spaw* expression. Interestingly, injection of either *prkci* MO or *prkcz* MO alone did not cause LR asymmetry defects, whereas injection of both *prkci* and *prkcz* MOs together did alter LR asymmetry. This indicates PrkCi and PrkCz function redundantly. As Pard3 functions with PrkCi and the function of Pard3 was only partially suppressed, we asked if morpholino suppression of both *pard3* and *prkci* would show an additive, or perhaps synergistic, effect on LR asymmetry. However, suppression of *pard3* and *prkci* did not alter the percentage of embryos showing left-sided expression of *spaw,* compared to suppression of *pard3* alone (Fig. 12B).

We performed immunocytochemistry on 8–10 somite stage embryos and labeled cilia with acetylated tubulin antibodies (Fig. 12C). While the size of the KV was similar between wild-type and *pard3* morphant embryos, the number of KV cilia was reduced (Fig. 12E) and cilia length was noticeably shorter (Fig. 12F). Taken together, the results suggest that Pard3 controls cilia formation in tissues other than the retina.

## Discussion

The purpose of this study was to investigate the role of the Par-PrkCi complex in cilia formation *in* *vivo*, with a specific emphasis on photoreceptor outer segment formation. The roles for the Par-PrkCi complex and Crb proteins as apical determinants has been well established [19]. From flies to humans the Crb proteins are required for photoreceptor morphogenesis, particularly in formation of the light-sensitive rhabdomeres or outer segments [28], [31], [48], [65]. This requirement reflects a role for Crb proteins in ciliogenesis [34]. While the Par-PrkCi complex was implicated in ciliogenesis in tissue culture [34], [35], it was unclear if such function occurred *in* *vivo*, particularly in photoreceptors. Bazooka (the *Drosophila* homolog of Pard3), PrkCi, and Par6 are required for targeting Crb to the rhabdomere stalk [26]. However, Crb also regulates rhodopsin trafficking through interactions with myosin V [66], thereby raising the possibility Crb role in ciliogenesis may be independent of Par-PrkCi. Consistent with this hypothesis, the loss of *prkci* or *pard3* in zebrafish clearly disrupted apico-basal polarity, but cilia defects were not previously reported [17], [38], [39], [51]. Nevertheless, a recent study reported that inhibition of the PrkCi ortholog in sea urchin embryos dramatically shortened cilia, suggesting a conserved role for this complex in ciliogenesis [67]. We report that inhibition of Pard3 or PrkCi/z activity impairs cilia formation.

### Role of *pard3* in apico-basal polarity

The loss of *pard3* impacts apico-basal polarity in two distinct events within the retina. Wei and colleagues [17] previously showed that a strong morpholino suppression of *pard3* disrupted retinal lamination and reduced photoreceptor numbers. The loss of *prkci* or *crb* produces similar phenotypes, suggesting that these genes function in a similar pathway [28], [38]. Based on observations in *Drosophila* and tissue culture, it is believed that Pard3 recruits PrkCi to cellular junctions, while Crb maintains junction integrity [21], [26]. Thus, the retinal lamination defect in strong *pard3* morphants likely reflects an initial requirement for *pard3* to establish apical cellular junctions within the developing neuroepithelium, similar to *prkci* [38]. The second role for *pard3* occurs in photoreceptors. Once photoreceptor cells acquire the appropriate laminar position, Pard3 and PrkC regulate the size of the apical domain. Suppression of *pard3* by sub-saturating doses of morpholinos or by the use of photo-morpholinos did not affect retinal lamination (Figs. 5, 8) but apical membrane size was reduced (Fig. 4, 8). Similarly, inhibition of PrkC activity by photo-morpholinos or by pharmacological inhibition resulted in a reduction of the apical membrane (Fig. 8, 10).

The precise mechanism by which Pard3 and PrkCi regulate apical size is unclear, but it likely involves the function of Crb proteins. Two *crb2* paralogs, *crb2a* and *crb2b*, are expressed in zebrafish photoreceptors. The loss of *crb2b* function in zebrafish photoreceptors reduced apical membrane size similar to our results with Pard3 and PrkC [28]. In contrast, overexpression of various domains of *crb2a* significantly increased the size of the apical membrane and outer segment [29]. Whether these protein complexes function together or in a sequential process remains poorly understood. Based on protein localization, it is unlikely that Pard3 participates in a complex with PrkCi or Crb to regulate apical membrane size. While Pard3 localizes to the OLM and the outer plexiform layer, PrkC and Crb localize immediately apical to the OLM and extend apically along the inner segment in a region that lacks Pard3. This is consistent with studies showing PrkC and Crb excluding Pard3 from the apical surface [23], [68]. It should be noted, however, that the formation of a Par-PrkC complex was required for apical membrane growth [23]. This suggests a sequential model where a temporary Par3-PrkC complex must assemble prior to PrkC migration to the apical surface [23]. Although modest suppression of Pard3 did not impact localization of PrkC or Crb to the region immediately apical to the OLM, we cannot exclude the possibility that some Pard3 activity remained.

### Role of *pard3* in cilia formation

As the ciliary axoneme grows during ciliogenesis, the ciliary membrane extends from the apical surface. The ciliary membrane is distinct from the apical cell membrane [69] and some cells form elaborate ciliary structures, such as the vertebrate photoreceptor outer segment. It is clear, however, that formation of the ciliary membrane requires the same apical determinants used to form the apical cell membrane. Manipulating *crb* expression can increase or decrease both apical and ciliary membranes [28], [29], while inhibition of Pard3 or PrkC similarly reduces apical size and leads to loss of cilia (this study). Previous work in zebrafish found that positioning of centrosomes and basal bodies within the early neural tube requires Pard3 [15]. We found normal basal body localization in photoreceptors following suppression of *pard3* by photo-MOs (data not shown), but this may reflect the early polarization of the retinal neuroepithelium.

The mechanism by which these apical determinants control ciliogenesis remains unclear. One possibility is that these proteins facilitate some aspect of intraflagellar transport (IFT). Work in tissue culture found that Pard3, PrkCi, and Crb3 localized in cilia and Pard3 and PrkCi immunoprecipitated with acetylated tubulin [34]. Pard3 can also directly bind to Kif3a, the anterograde motor for IFT [55]. In contrast, numerous studies in *Drosophila* and mammalian cells have found Pard3 localizing to a subapical domain in a pattern distinct from PrkCi and Par6 [19], [23], [26]. We failed to observe colocalization of Pard3 with acetylated tubulin in the connecting cilia of photoreceptors and Pard3 did not localize along the apical surface with PrkC or Crb. While we cannot exclude the possibility that ciliary localization of Pard3 escaped our detection, our results are consistent with previous studies where a different polyclonal antibody did not detect Pard3 in photoreceptor cilia [17], and those showing a Pard3-GFP fusion protein not localizing to cilia when expressed within the zebrafish neural tube [15]. Given the distinct localization patterns between Pard3 and other apical determinants, our results favor a mechanism whereby Pard3 regulates the size of the entire apical membrane. While the ciliary and non-ciliary compartments have distinct protein profiles [69], cilia formation typically requires prior specification of the apical membrane.

The apical determinant factors *pard3, prkci, nok, moe, and ome* all give similar retinal phenotypes when fully mutated or suppressed, yet cilia defects in other tissues (e.g. kidney, otic vesicle, Kupffer’s Vesicle) are not always observed [17], [28], [39], [49], [50]. We observed defects in left-right asymmetry and KV cilia in *pard3* morphants and hydrocephalus was previously noted [15], although kidney cysts were not observed. In many cases, however, genetic redundancy by paralogs can compensate for the loss of individual genes. With *crb* genes, for example, *ome* functions in neuroepithelia, *crb2b* functions in the retina and kidney, while *crb3a* functions in the otic vesicle [28]. Similarly, *prkci* and *prkcz* exhibit functional redundancy within the retina [38]. A *pard3b* paralog [70], may explain why *pard3* morphants do not exhibit other ciliary defects.

We used three distinct experimental approaches to inhibit Pard3 and PrkC activity and all gave similar results. Sub-optimal concentrations of morpholinos permitted retinal lamination in *pard3* morphants and partially abrogated cilia and outer segment formation. A well-established peptide inhibitor of PrkC was effective in reducing apical domain size, but the inhibitor was toxic at earlier time points, which likely limits its utility for studies in embryos. The use of photo-morpholinos gave the strongest effects, particularly when targeting the *prkc* genes. While these reagents did not completely inhibit the MO activity over several days, the ability to cleave the photo-MO provided temporal control over MO activity and permitted normal retina lamination with before suppression of either *pard3* or *prkc*. It should be noted that photo-morpholino suppression of *prkci* or *prkci/z* resulted in much more severe retinal effects than suppression of *pard3*. Although a biological difference cannot be definitively ruled out, these observations may reflect a difference in the efficacy of photo-MOs at different gene targets. While Tallafuss and colleagues reported strong suppression with 5–10 minutes of UV irradiation at 5 hpf [40], at least 20 minutes of irradiation was required to suppression gene function in our studies at either 5 hpf or 30 hpf. Longer irradiation times (e.g. 30 minutes) did not enhance the phenotype, although it is possible that the *pard3* photo-MO was still not completely cleaved. As with any antisense reagent, the efficacy of photo-MOs can vary from target to target and this may explain the differences in phenotype.

In summary, we show that cilia formation in vertebrate photoreceptors requires the Par-PrkC complex in a manner that likely reflects a role in apical membrane regulation. Mutations in *CRB1* cause retinitis pigmentosa [65] and *crb* genes were known to play key roles in apical differentiation. We now show that cilia formation *in* *vivo* requires the involvement of the Par-PrkC complex as well.

## Supporting Information

Figure S1 **Immunoblot of Pard3 from retinal homogenate.** Retinal homogenates at 1∶50 or 1∶200 dilutions were separated on SDS-PAGE incubated with anti-Pard3 antiserum. A immunoreactive band was seen at ∼180 kDa. A second, higher molecular weight band was nonspecific. BioRad molecular weight ladder (in kDa) is shown to the left.
(TIF)

Figure S2 **Genetic analysis of photoreceptor formation at 96 hpf.** (A) Wild-type donor cells with rhodamine-dextran lineage label (pseudo-colored green) in a wild-type host retina. (B) *pard3-*morphant donor cells in a wild-type host retina. Only one photoreceptor formed from donor cells. (C) *prkci*-morphant donor cells in a wild-type host retina. Two photoreceptors were formed by donor cells. (D) *prkci/prkcz*-double morphant donor cells in a wild-type host retina. No donor cells contributed to the photoreceptor layer. All sections were counterstained with DAPI (blue). Scale bar = 10 µm.
(TIF)

Figure S3 **Assessing photo-morpholino efficacy by morphological phenotypes at 3 dpf following UV irradiation.** (A, B) wild type larvae with and without UV irradiation, (C) *pard3* morphant, (D) *prkci* morphant, (E, F) larvae injected with *pard3* or *prkci* MO/S-photo-MOs hybrids without UV irradiation, (G, H) larvae injected with *pard3* or *prkci* MO/S-photo-MOs hybrids and irradiated with UV light at 5 dpf, (I, J) larvae injected with *pard3* or *prkci* MO/S-photo-MOs hybrids and irradiated with UV light at 30 hpf, and (K, L) larvae injected with *pard3* or *prkci* S-photo-MOs only. When S-photo-MOs were cleaved by UV light irradiation at 5 hpf, embryos showed similar phenotypes (e.g. body curvature problems, patchy ocular pigmentation, reduced eye size, similar to the *pard3-*MO or *prkci* morphants (compare panels E and F to panels G and H). Scale bar = 500 µm.
(TIF)

Figure S4 **Photo-morpholinos inhibit the action of morpholinos in the absence of UV irradiation and preserve retinal structure.** (A–F) Transverse cryosections of 3 dpf retinas were stained with phalloidin to label actin (red) and DAPI to label nuclei (blue). The top row shows retinal organization in various larvae following UV-irradiation at 5 hpf while the bottom row is without UV irradiation. (A, D) Wild type; (B, E) *pard3* MO/S-photo-MO hybrid; (C, F) *prkci* MO/S-photo-MO hybrid. Retinal architecture is disrupted and the plexiforms layers more disorganized when photo-MOs are cleaved by UV irradiation (top row). Retinal lamination is largely preserved when photo-MOs remain intact in the absence of UV irradiation, indicating inhibition of morpholino action (bottom row). Scale bar = 100 µm.
(TIF)
