# Supplementary figures and images for: The Par-PrkC Polarity Complex Is Required for Cilia Growth in Zebrafish Photoreceptors
Source: PLoS One. 2014 Aug 21;9(8):e104661. doi: 10.1371/journal.pone.0104661 (PMC4140697; doi:10.1371/journal.pone.0104661)

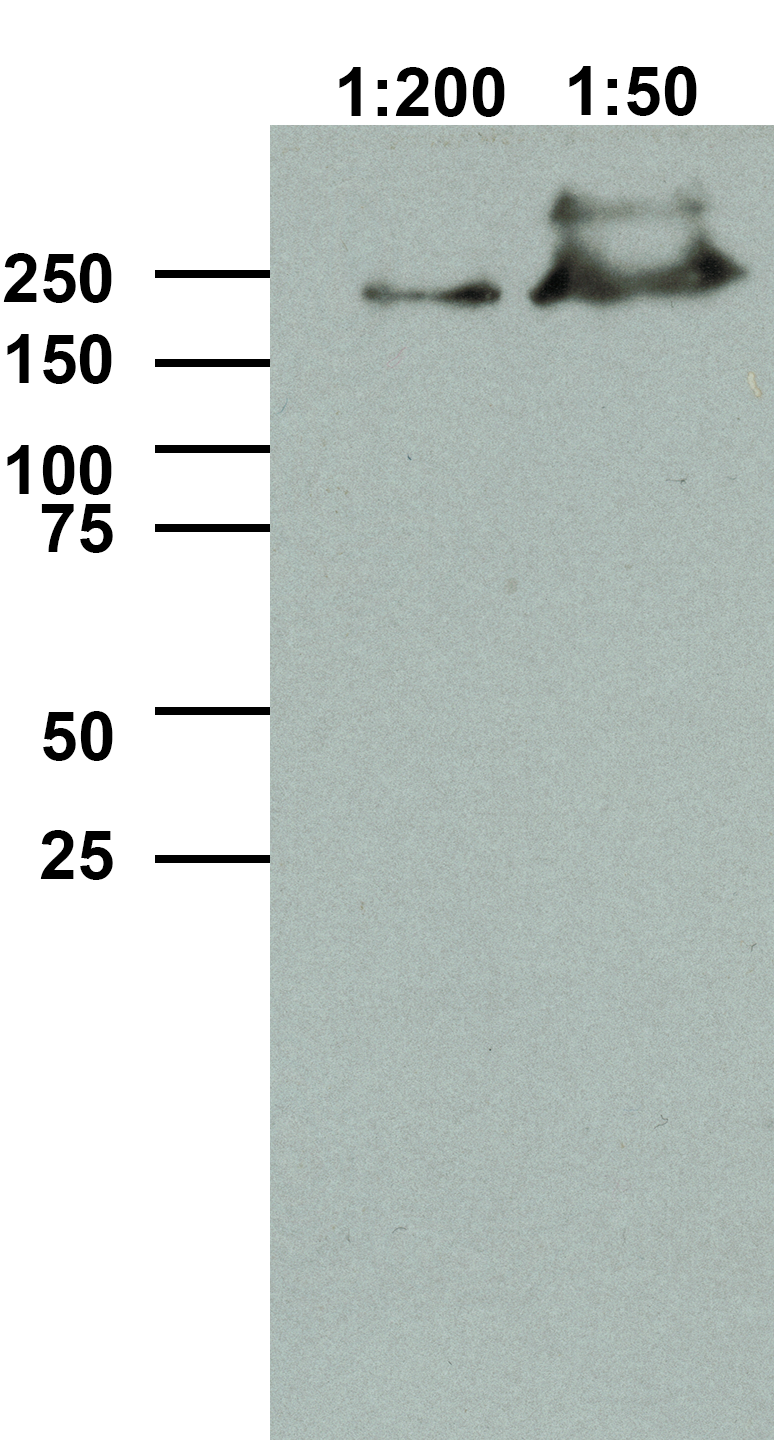

Supplement: Figure S1 — Immunoblot of Pard3 from retinal homogenate. Retinal homogenates at 1∶50 or 1∶200 dilutions were separated on SDS-PAGE incubated with anti-Pard3 antiserum. A immunoreactive band was seen at ∼180 kDa. A second, higher molecular weight band was nonspecific. BioRad molecular weight ladder (in kDa) is shown to the left. (TIF) [file pone.0104661.s001.tif]

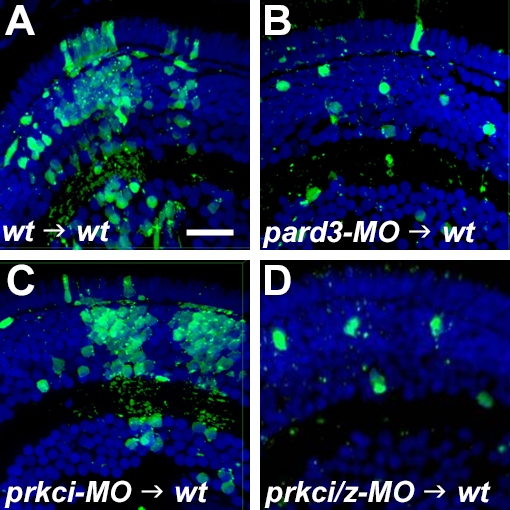

Supplement: Figure S2 — Genetic analysis of photoreceptor formation at 96 hpf. (A) Wild-type donor cells with rhodamine-dextran lineage label (pseudo-colored green) in a wild-type host retina. (B) pard3-morphant donor cells in a wild-type host retina. Only one photoreceptor formed from donor cells. (C) prkci-morphant donor cells in a wild-type host retina. Two photoreceptors were formed by donor cells. (D) prkci/prkcz-double morphant donor cells in a wild-type host retina. No donor cells contributed to the photoreceptor layer. All sections were counterstained with DAPI (blue). Scale bar = 10 µm. (TIF) [file pone.0104661.s002.tif]

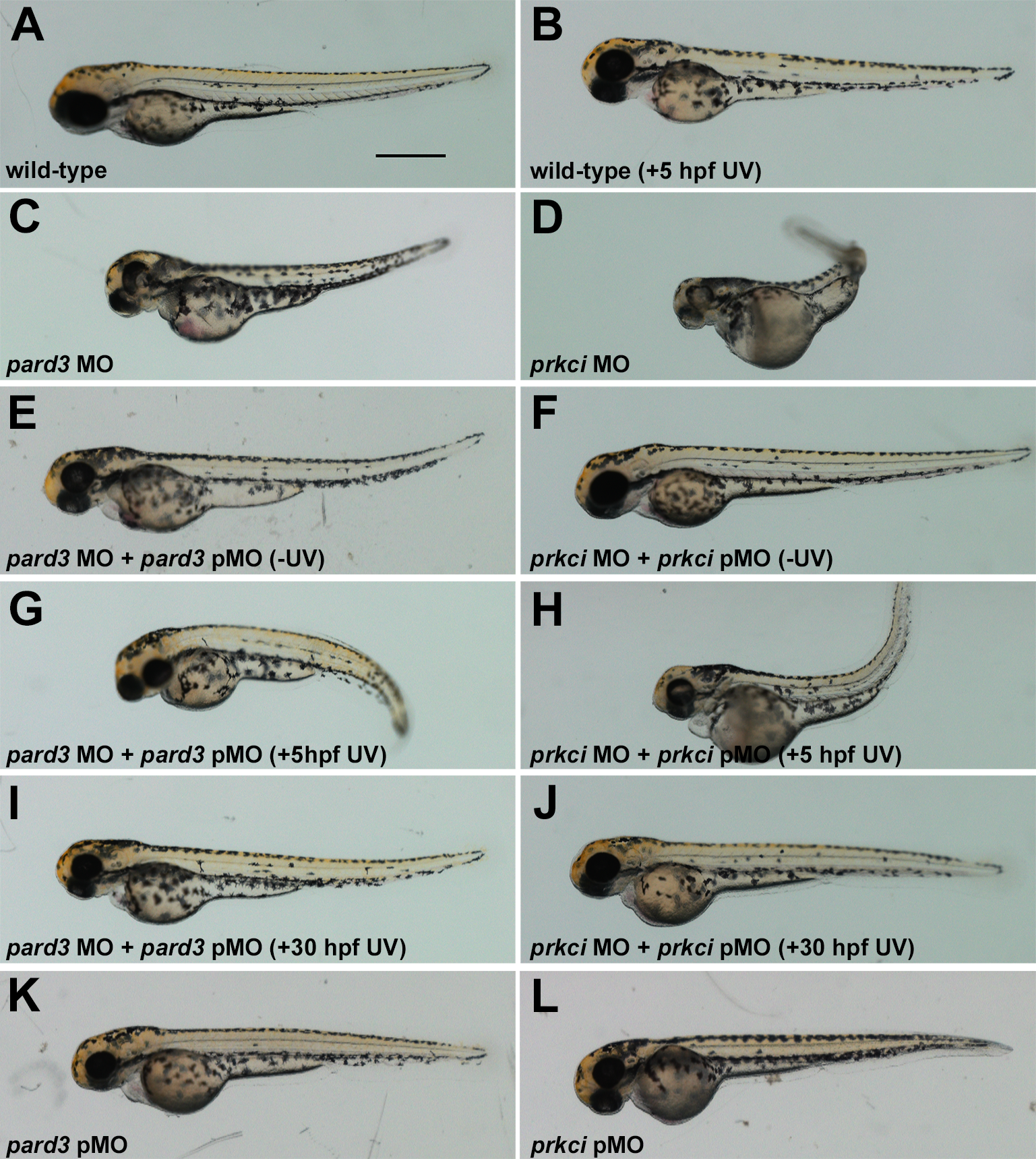

Supplement: Figure S3 — Assessing photo-morpholino efficacy by morphological phenotypes at 3 dpf following UV irradiation. (A, B) wild type larvae with and without UV irradiation, (C) pard3 morphant, (D) prkci morphant, (E, F) larvae injected with pard3 or prkci MO/S-photo-MOs hybrids without UV irradiation, (G, H) larvae injected with pard3 or prkci MO/S-photo-MOs hybrids and irradiated with UV light at 5 dpf, (I, J) larvae injected with pard3 or prkci MO/S-photo-MOs hybrids and irradiated with UV light at 30 hpf, and (K, L) larvae injected with pard3 or prkci S-photo-MOs only. When S-photo-MOs were cleaved by UV light irradiation at 5 hpf, embryos showed similar phenotypes (e.g. body curvature problems, patchy ocular pigmentation, reduced eye size, similar to the pard3-MO or prkci morphants (compare panels E and F to panels G and H). Scale bar = 500 µm. (TIF) [file pone.0104661.s003.tif]

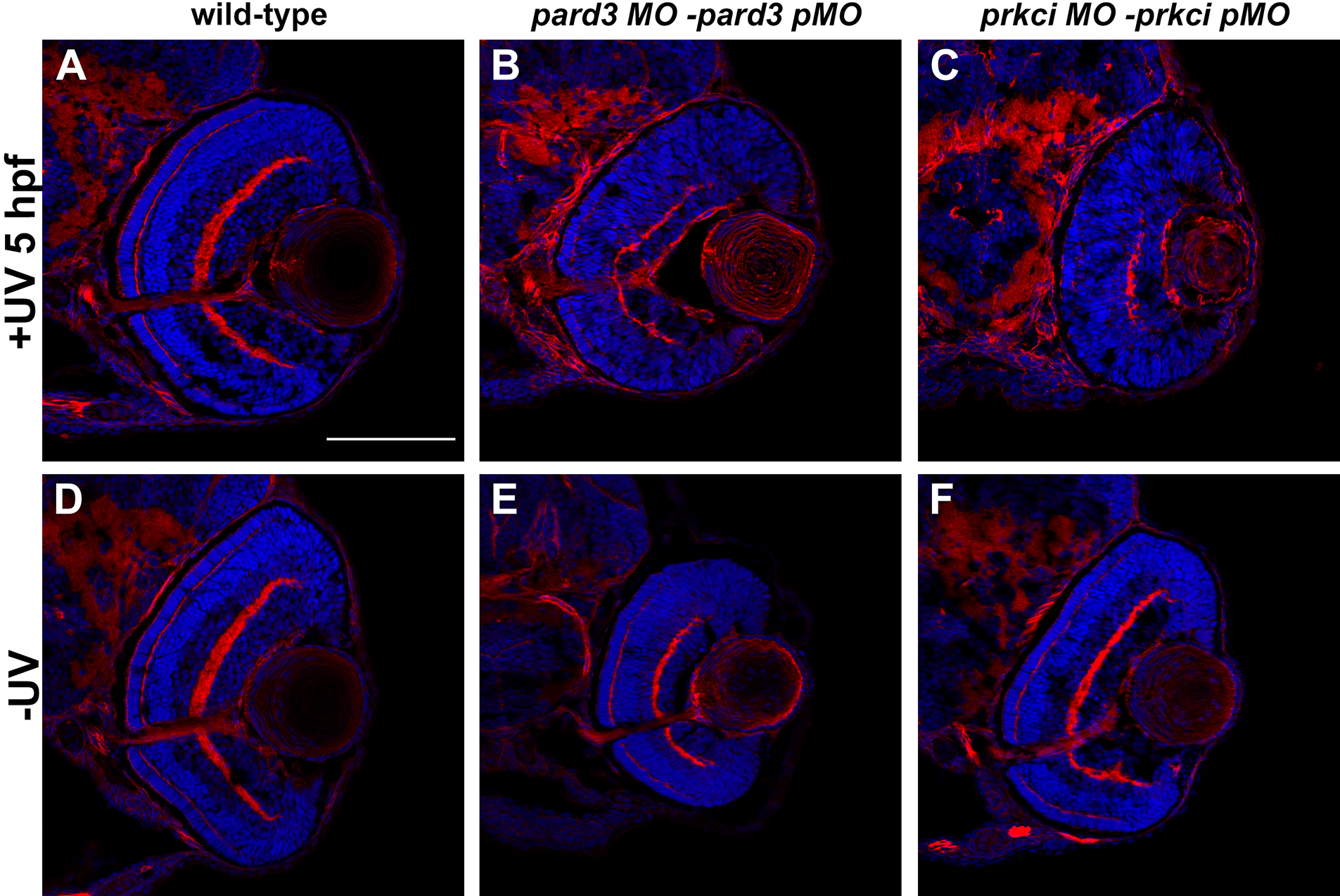

Supplement: Figure S4 — Photo-morpholinos inhibit the action of morpholinos in the absence of UV irradiation and preserve retinal structure. (A–F) Transverse cryosections of 3 dpf retinas were stained with phalloidin to label actin (red) and DAPI to label nuclei (blue). The top row shows retinal organization in various larvae following UV-irradiation at 5 hpf while the bottom row is without UV irradiation. (A, D) Wild type; (B, E) pard3 MO/S-photo-MO hybrid; (C, F) prkci MO/S-photo-MO hybrid. Retinal architecture is disrupted and the plexiforms layers more disorganized when photo-MOs are cleaved by UV irradiation (top row). Retinal lamination is largely preserved when photo-MOs remain intact in the absence of UV irradiation, indicating inhibition of morpholino action (bottom row). Scale bar = 100 µm. (TIF) [file pone.0104661.s004.tif]
